# Conformations of Steroid Hormones: Infrared and Vibrational Circular Dichroism Spectroscopy

**DOI:** 10.3390/molecules28020771

**Published:** 2023-01-12

**Authors:** Yanqing Yang, Anna Krin, Xiaoli Cai, Mohammad Reza Poopari, Yuefei Zhang, James R. Cheeseman, Yunjie Xu

**Affiliations:** 1 Department of Chemistry, University of Alberta, Edmonton, AB T6G 2G2, Canada; 2 Centre for Science and Peace Research (ZNF), Universität Hamburg, Bogenallee 11, 20144 Hamburg, Germany; 3 Key Laboratory for Green Chemical Process of Ministry of Education, Wuhan Institute of Technology, Wuhan 430073, China; 4 Gaussian Inc., 340 Quinnipiac St., Bldg., 40, Wallingford, CT 06492-4050, USA

**Keywords:** IR spectroscopy, vibrational circular dichroism, steroid hormones, absolute configuration and conformation

## Abstract

Steroid hormone molecules may exhibit very different functionalities based on the associated functional groups and their 3D arrangements in space, i.e., absolute configurations and conformations. Infrared (IR) and vibrational circular dichroism (VCD) spectra of four different steroid hormones, namely dehydroepiandrosterone (DHEA), 17*α*-methyltestosterone (MTTT), (16α,17)-epoxyprogesterone (Epoxy-P4), and dehydroepiandrosterone acetate (AcO-DHEA), were measured in deuterated dimethyl sulfoxide and some also in carbon tetrachloride. Extensive conformational searches were carried out using the recent developed conformer-rotamer ensemble sampling tool (CREST) which also accounts for solvent effects using an implicit solvation model. All the CREST conformational candidates were then reoptimized at the B3LYP-D3BJ/def2-TZVPD with the PCM of solvent. The good agreements between the experimental IR and VCD spectra and the theoretical simulations provide a conclusive information about their conformational distribution and absolute configurations. The experimental and theoretical IR and VCD spectra of AcO-DHEA in the carbonyl and alkene stretching region showed some discrepancies, and the possible causes related to solvent effects, large amplitude motions and levels of theory used in the modelling were explored in detail. As part of the investigation, additional calculations at the B3LYP-D3BJ/6-31++G (2d,p) and B3LYP-D3BJ/cc-pVTZ levels, as well as some ‘mixed’ calculations with the double-hybrid functional B2PLYP-D3 were also carried out. The results indicate that the double-hybrid functional is important for predicting the correct IR band pattern in the carbonyl and alkene stretching region.

## 1. Introduction

**Scheme 1:**
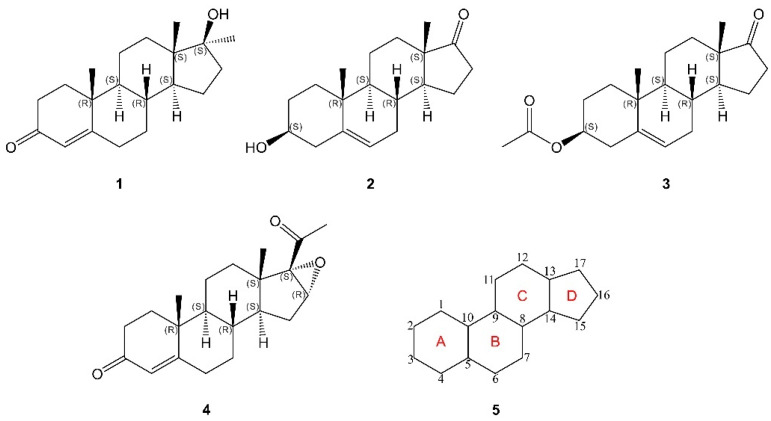
Structure of four steroid compounds studied in this paper and their common nucleus core: **1**: methyltestosterone (MTTT), **2**: dehydroepiandrosterone (DHEA), **3**: acetylated dehydroepiandrosterone (AcO−DHEA), **4**: (16*α*,17)−epoxyprogesterone (Epoxy−P4). **5**: ‘Steroid nucleus’: the carbon skeleton of the parent compound (gonane) of the steroids. The atomic numbers are given in **5**. The comparison with some other major steroids is given in Scheme S1.

Steroid hormones play a crucial role in multiple signaling pathways in living organisms, cementing their great importance in biology, medicine, and pharmacology [1,2]. Despite their functional diversity, steroid hormones usually pose some structural similarities. They are cholesterol derivatives and share the same core structure called gonane, which consists of three cyclohexane rings (A, B, C) and one cyclopentane ring (D) fused together (Scheme 1) [3]. Some steroid hormones differ only in the functional groups attached to this core structure. To appreciate the large differences in the resulting biological effects caused by these seemingly minor structural variations, it is important to gain detailed structural characterization of these steroids. Additionally, it should be emphasized that steroids are chiral molecules and may lead to subsequent enantioselectivity in the human body. For example, enantiomers of neuroactive steroids such as pregnanolone and allopregnanolone differ in their ability to activate and to inhibit the so-called GABA-C receptors [4]. An unambiguous characterization of the absolute configuration and conformation of a steroid candidate is therefore crucial to ensure its proper enantioselective interaction inside human body.

X-Ray diffraction has been widely used to obtain the absolute configurations of chiral compounds with different chemical compositions [5,6]. One main shortcoming is that a high-quality single crystal of the analyte is required, a condition which may not be possible in some cases. Recently, several new techniques have been developed for structural characterization of chiral molecular systems in the gas phase. These include, for example, Coulomb explosion imaging [7,8], the microwave three-wave mixing technique [9], chiral-tag rotational spectroscopy [10,11], and photoelectron circular dichroism (PECD) [12,13]. Chiroptical spectroscopies, on the other hand, can provide significant insight into structural information including absolute configurations and conformational distributions of chiral molecules directly in solution. While optical rotation (OR) [14] and especially electronic CD (ECD) [15] have been utilized for determination of absolute configurations, decisive comparisons with the corresponding theoretical predictions may be hampered by serious solvent effects in OR [16,17] and broad band width in ECD, as well as challenges in theoretical simulations of OR and ECD. In addition, ECD requires a chromophore that absorbs in the UV-vis region, which may be lacking in many chiral molecules. These limitations can be generally overcome by using vibrational circular dichroism (VCD) and/or Raman optical activity (ROA) spectroscopy; the former is broadly used as an analytical chemistry tool in pharmaceutical industry.

In the current study, we utilized VCD spectroscopy which measures the difference in the absorption intensity of the left versus right circularly polarized light when a molecule undergoes a vibrational transition. Initially developed in 1970s [18], VCD has experienced significant advances in both experimental and theoretical aspects [19,20,21]. The absolute configuration determination by VCD with the aid of the density functional theory (DFT) calculations has been successfully applied to a wide range of molecules in solution, for example, natural products and herbal products [21,22,23], peptides and proteins [24,25], and transition metal complexes [26,27], just to name a few. In addition, there have been considerable literatures on how to effectively account for solvent effects in VCD measurements [28,29,30], and such considerations further improve the agreement between experiment and theory in general.

We carried out a combined experimental and theoretical investigation of four steroid hormones (see Scheme 1) in solution by using both IR and VCD spectroscopy to elucidate their structural properties including absolute configurations and conformational distributions. One of the compounds studied is dehydroepiandrosterone (DHEA) which is used as a precursor in synthesis of sexual hormones [31]. DHEA is also shown to have a variety of biological effects in the human body, including antidepressant and antitumor effects [32,33]. Next, the acetylated form of DHEA, dehydroepiandrosterone acetate (AcO-DHEA) was also studied. Similar to many biological specimens, derivatization of steroids with acetyl or trimethylsilyl groups is typically required before they can be analyzed using gas chromatography (GC) or liquid chromatography (LC) with mass spectrometry [34]. It would be of considerable interest to compare the conformational preferences of DHEA and AcO-DHEA from the IR and VCD spectroscopic data. Progesterone is another well-known endogenous steroid and plays an important role in reproductivity and embryogenesis [35] and in regulating immune response in newborns [36]. The third compound studied is a derivative of progesterone, namely (16*α*, 17)-epoxy progesterone (Epoxy-P4), an important intermediate in the production of steroidal drugs [37]. Because of their significance to our health, many steroid hormones are nowadays synthetically produced and used as medication for treating a wide range of health issues. The commercially available synthetic analogs of testosterone belong to this group. These analogs have usually an alkyl group substituent in order to improve their oral bioavailability. The fourth compound investigated is a testosterone analog belonging to this group, namely methyltestosterone, i.e., 17*α*-methyltestosterone (MTTT), which bears a methyl group at the carbon atom C-17. MTTT has been broadly used in aquafarming and more recently concerns have been raised about its ecological risk [38].

One objective of this paper is to directly compare the similarities and differences of the experimental IR and VCD spectra of these steroid hormones in solution to identify markers for specific functional groups. The importance of such markers was illustrated in a situation where these marker features were used to reveal the identity of a mislabeled steroid compound in the current study. Second, extensive theoretical conformational searches and DFT calculations with different functionals and basis sets were carried out to facilitate theoretical IR and VCD simulations and comparisons with experiment. We aimed to investigate the influence of conformational distributions and absolute configurations of these steroid hormones on the resulting spectral features. Third, we examined structural flexibility around rotatable bonds in these steroids and evaluated their effects on the resulting IR and VCD spectra. Often multiple minima were identified theoretically associated with the rotatable bonds. In some cases, these minima turned out to be unstable after zero-point-energy (ZPE) correction, i.e., when the ZPE level is higher than the related conformational conversion transition state. In recent rotational spectroscopic studies of 1-phenyl-2,2,2-trifluoroethanol (PhTFE) [39] and its complex with a water molecule [40], PhTFE-W, it was reported that several DFT minima identified do not correspond to true conformers, i.e., they are connected by the so-called large amplitude motions. Furthermore, in those cases, some experimentally observed ground state properties, such as dipole moment components, deviate drastically from those predicated at the minima. These gas phase studies highlight the potential issues with the common practice of generating IR and VCD spectra based on the DFT minima. These issues will be addressed in the discussion of the IR and VCD spectra of AcO-DHEA. Fourth, some disagreements between experimental and theoretical IR and VCD were noted for AcO-DHEA in the carbonyl and alkene stretching region and the possible causes related to solvent effects, large amplitude motions and theoretical levels of theory were explored in detail.

## 2. Results and Discussion

In the following, we describe how IR and other supporting techniques were used to identify a mislabeled sample in Section 2.1. The experimental IR and VCD features of the four compounds are compared and commented in Section 2.2. Then we describe the general conformational searches and DFT geometry optimizations performed for each compound and summarize all the low energy minima identified in Section 2.3. Subsequently, comparisons of the simulated and experimental IR and VCD spectra are discussed for MTTT, DHEA and Epoxy-P4 in Section 2.4. In Section 2.5, we address the challenges in the IR and VCD simulations of AcO-DHEA in the >1700 cm^−1^ region. Three possible causes related to solvent effects, large amplitude motions and levels of theory used in the modelling were explored in detail to understand the disagreement between the simulated IR and VCD patterns and the experimental ones in this particular wavenumber region. Overall, the good agreements between experiment and theory allow one to conclusively identify the main conformers in solution. Finally, some brief comments on the absolute configurations of the four steroids are made in Section 2.6.

### 2.1. Using Experimental IR and VCD Spectra to Identify a Mislabeled Sample

**Figure 1:**
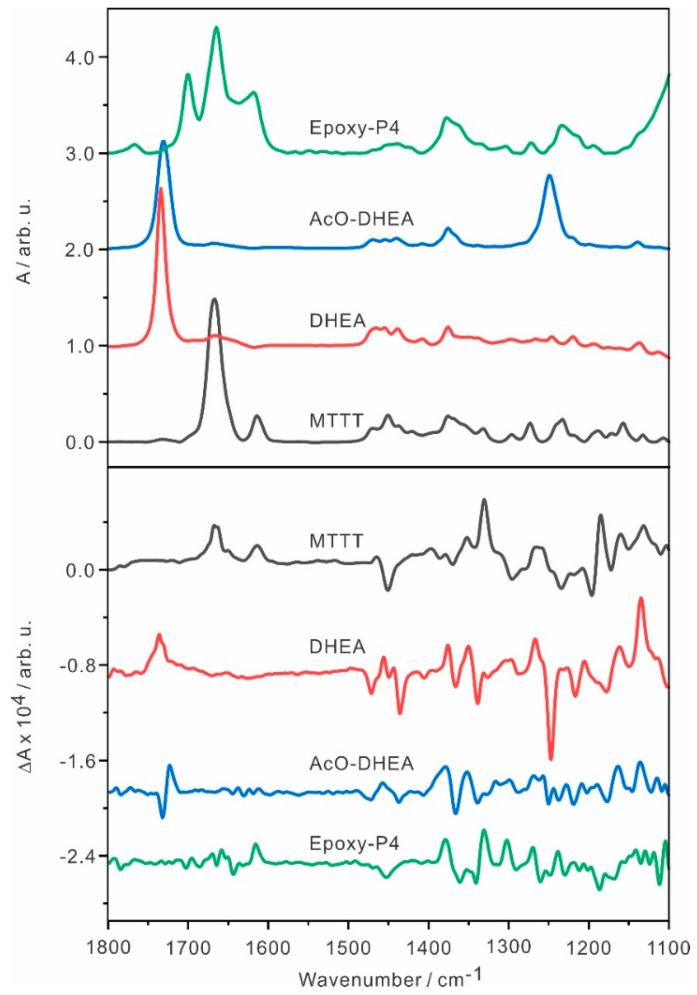
Experimental IR and VCD spectra of MTTT, DHEA, AcO−DHEA and Epoxy−P4 measured in DMSO−*d*_6_.

Initially, two steroid samples labelled as ‘MTTT’ and DHEA were received. Their experimental IR spectra were measured in DMSO-*d*_6_ and shown in Figure S1, Supplementary Material, together with the corresponding theoretical IR spectra of the most stable conformers of MTTT and DHEA (vide infra). There was much confusion about the sample labelled as ‘MTTT’. First, DHEA shows a strong IR band at 1733.9 cm^−1^, while the ‘MTTT’ sample exhibits a strong and somewhat broad IR feature centered at 1730.5 cm^−1^. These features were tentatively assigned to be associated with the C=O stretch in each compound. On the other hand, this assignment is inconsistent with the expectation and the related DFT calculations, i.e., the C=O stretching band of MTTT should appear at a somewhat lower wavenumber than that of DHEA because of the hyperconjugation of the C=O bond with the C=C bond in MTTT. Second, the experimental ‘MTTT’ VCD exhibits a bisignate feature at the C=O stretching band (Figure 1), seemingly suggesting that there are two C=O groups in the molecule, in contrast with the MTTT geometry shown in Scheme 1. Third, the ‘MTTT’ IR spectrum we obtained appears very different when compared to the solid IR spectrum of MTTT (nujol mull) reported on the National Institute of Standards and Technology (NIST) website [41]. From all these experimental evidences, we concluded that the sample labelled as ‘MTTT’ is *not* MTTT.

What could the mysterious ‘MTTT’ sample be? There were speculations of oxidation and decomposition of the sample and/or some unusual hydrogen bonding interactions with the DMSO-*d*_6_ solvent. We set out to test each of these hypotheses. First, IR and VCD experiments using a non-polar solvent, CCl_4_, were also performed. The resulting spectra, which are shown in Figure S2, are very similar to those obtained in DMSO-*d*_6_ indicating that there is no drastic solvent effect here. Next, the standard GS-MS test of the ‘MTTT’ sample was performed and the result (see Figure S3) showed a single peak, confirming that the sample had not decomposed or been modified. The ESI-MS run gave a mass of 331 amu, definitely not MTTT whose mass is 302 amu. Extensive literature searches led us to the IR spectrum of 7-oxo-DHEA [42] (see Figure S1), a molecule which contains three C=O groups and one C=C group. The IR spectrum of 7-oxo-DHEA exhibits very similar IR band features to our mysterious compound, except the extra C=O at 1673 cm^−1^. This comparison and the experimental mass value led us to propose AcO-DHEA as the possible identify of the mystery sample. Afterwards, we obtained additional samples of AcO-DHEA and MTTT from the company and were able to conclusively verify our identification. We note that AcO-DHEA is an intermediate product of both MTTT and DHEA.

### 2.2. Experimental IR and VCD Spectra of the Four Steroids in DMSO

The experimental IR and VCD spectra of MTTT, DHEA, AcO-DHEA and Epoxy-P4 measured in DMSO-*d*_6_ are presented in Figure 1. Both DHEA and AcO-DHEA have six stereogenic centers and share the same main molecular frame. While DHEA has a hydroxyl group substitute at C3, AcO-DHEA has an ester group at C3. Not surprisingly, their experimental IR spectra exhibit the anticipated similarity, except the additional strong band at ~1249 cm^−1^ for AcO-DHEA. This strong band can be attributed to the highly polar C-O stretch of the ester group of AcO-DHEA. In addition, AcO-DHEA has two carbonyl groups: one locates at the C17 position of the five-membered ring, and the other locates at the acetyl group. It appears that these two C=O stretching bands overlap strongly in the experimental IR spectrum, leading to only one slightly broadened carbonyl band at ~1730 cm^−1^. Interestingly, the situation results in a bisignate VCD signature for AcO-DHEA at the C=O stretching region, whereas only one positive VCD feature (at ~1736 cm^−1^) was observed in the same wavenumber region for DHEA. Moreover, some obvious VCD band pattern differences are present in the 1100–1300 cm^−1^ region.

MTTT also has six stereogenic centers and differs from DHEA in these three main aspects: (1) The C=C bond moves from ring B (C5=C6) in DHEA to ring A (C4=C5) in MTTT. The C=O stretching frequency in MTTT is significantly red shifted from that of DHEA because of its proximity to the C=C bond (at 1613 cm^−1^), occurring at ~1667 cm^−1^ instead of ~1731 cm^−1^; (2) The keto group at C17 in DHEA is replaced by a hydroxyl and a methyl group in MTTT; (3) The C=O group at C3 in MTTT is replaced by OH in DHEA. Except for some common weak bands in the 1350–1500 cm^−1^ region, MTTT and DHEA exhibit very different IR and VCD features. On the other hand, MTTT and Epoxy-P4 share largely the same structural motifs, except the substitutes at the five-membered ring, D, where the hydroxy and methyl group substitutes at C17 are replaced with an acetyl and an epoxy group where the latter is also bonded at C16. While Epoxy-P4 exhibits the C=O (at 1665 cm^−1^) and C=C (at 1620 cm^−1^) stretching bands at similar frequencies as those in MTTT, an additional C=O stretch of the acetyl group of Epoxy-P4 is noted at a higher frequency of ~1700 cm^−1^. Since VCD features are highly sensitive to small structural differences, there are no obvious similarity in the VCD spectra of these four steroids, even though some similarities in their parent IR spectra are observed.

### 2.3. Conformational Searches and Identification of Low Energy Minima of the Four Steroids

All four steroid molecules contain a similar fused ring core structure. While MTTT and Epoxy-P4 share the same fused ring core with A (cyclohexene), B and C (cyclohexane) and D (cyclopentane), DHEA and AcO-DHEA have the same core with A and C (cyclohexane), B (cyclohexene) and D (cyclopentanone). One may anticipate that the conformational distributions of these steroids arise mainly from the flexible substituents at these rings and also from the ring conformations. As the first step, systematical conformational searches were performed to identify the most stable minima of the four steroids.

**Figure 2:**
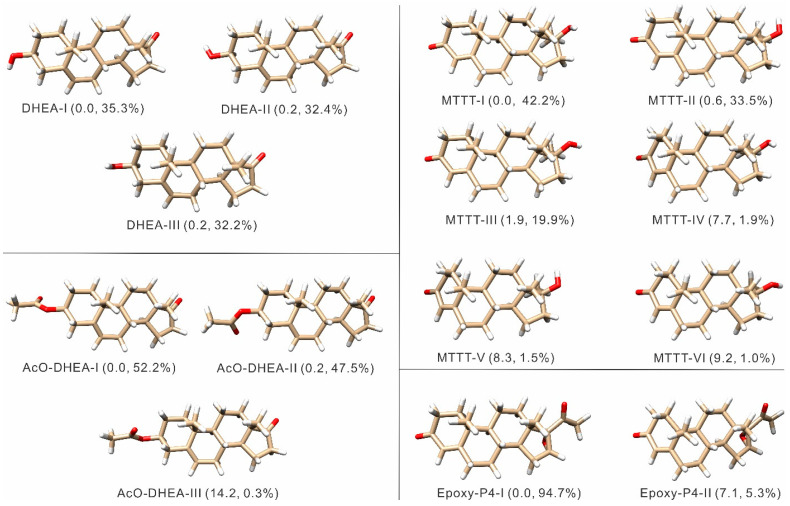
Optimized geometries of the conformers of the four steroids at the B3LYP−D3BJ/def2−TZVPD level of theory with the PCM of DMSO. The relative free energies in kJ mol^−1^ and the Boltzmann percentage abundances at 298 K are provided in brackets.

We chose to use the newly developed fast conformer-rotamer ensemble sampling tool, CREST [43]. CREST has been utilized and benchmarked extensively by us and others in rotational spectroscopic studies of a wide range of molecules and non-covalently bonded clusters [44,45,46] and in IR chirality recognition studies of protonated amino acid binary aggregates [47]. Generally, the experimentally observed conformers were all identified in the CREST searches. One exception reported recently is the weakly bound heterochiral trimer of propylene oxide [48] where the most stable geometry established experimentally is not a minimum in CREST. Another advantage of CREST is the readily available implicit solvation models for different solvents which can be used during a conformational search. We used DMSO for MTTT, DHEA, AcO-DHEA, and Epoxy-P4 conformational searches. Some additional loose geometry optimizations and single point energy evaluation steps are described in Section 3, Materials and Methods, as well as other CREST details. In total, 12 AcO-DHEA, eight DHEA, 10 MTTT and four Epoxy-P4 candidates were generated after the initial CREST searches. The final geometry optimizations were carried out at the B3LYP [49,50]-D3BJ/def2-TZVPD [51], B3LYP-D3BJ/6-31++G(2d,p) [52], and/or B3LYP-D3BJ/cc-pVTZ [53] levels of theory with the polarizable continuum model (PCM) [54] of DMSO. The D3 dispersion correction [55,56] with the Becke–Johnson (BJ) damping function [57] was used. This procedure generated six MTTT, three AcO-DHEA, three DHEA, and two Epoxy-P4 conformers within an energy window of 10 kJ mol^−1^. The corresponding optimized geometries are depicted in Figure 2, together with their relative free energies and their Boltzmann percentage abundances at 298 K.

For the most stable DHEA and AcO-DHEA conformers shown in Figure 2, they all share the same steroid nucleus geometry. Ring A, a cyclohexane ring, takes on the chair conformation with the OH (or AcO in the AcO-DHEA) and the CH_3_ substitute occupying the equatorial versus axial position, respectively. Ring B, a cyclohexene ring, exhibits a half-chair conformation [58,59], whereas Ring C (cyclohexane) again takes on a chair conformation. Next, the cyclopentanone ring, Ring D, takes on the ‘twisted’ conformation with slight distortion because of the neighboring cyclohexane fused ring. We note that the isolated cyclopentanone was established to be in a twisted conformation with a *C*_2_ symmetry axis and the barrier to pseudorotation in fairly high [60,61], in contrast to that in cyclopentane [62]. Overall, for these six conformers, the cyclohexane and cyclohexene rings appear to adopt the same most stable conformations in their respective isolated monomers [63,64].

The core structures with the MTTT and Epoxy-P4 conformers in Figure 2 differ somewhat from those discussed above. First, the cyclopentane ring, Ring D, takes on the well-known ‘envelope’ conformation with four C atoms in the same plane and one out of plane, denoted as the *endo* C. The situation in the isolated cyclopentane molecule is somewhat complicated: while the envelope conformation with a *C_s_* symmetry is the minimum, the pseudorotation through the half-chair maximum has only a barrier of 0.5 kcal mol^−1^ [62,65]. Not surprisingly, the preference for the envelope versus half-chair can be easily altered depending on the substituents introduced and even the solvents used [62]. The preference for the envelope conformation in MTTT and Epoxy-P4 is reinforced by the neighboring fused cyclohexane ring C. Second, the cyclohexane rings, i.e., B and C, take on the chair conformation as anticipated. Finally, A (cyclohexene ring) takes on the half-chair conformation as expected, except in the case of the much less stable MTTT-IV and V and Epoxy-P4-II where noticeable distortion to the half-chair conformation was noted. Please see Figure S4, for the comparison of MTTT-I and IV in this regard.

Besides the fused ring conformations, the conformational differences mainly arise from the flexibility of the substituents. For example, in the cases of MTTT and DHEA, the relative orientation or pointing direction of the OH group generates three different conformers I, II and III in each case. For AcO-DHEA, the rotation of the AcO group about the O-C bond attached to the ring gives rise to I, II, and III conformers, whereas rotation of the C=O group about the C-O bond appears to be somewhat hindered because of some degree of resonance between C=O and the connected C-O. In the case of Epoxy-P4, the epoxy and the ketone substitutes seem to be locked in place and the two conformations I and II are due to the A ring conformations, as discussed above.

### 2.4. Experimental and Simulated IR and VCD Spectra of DHEA, MTTT and Epoxy-P4

**Figure 3:**
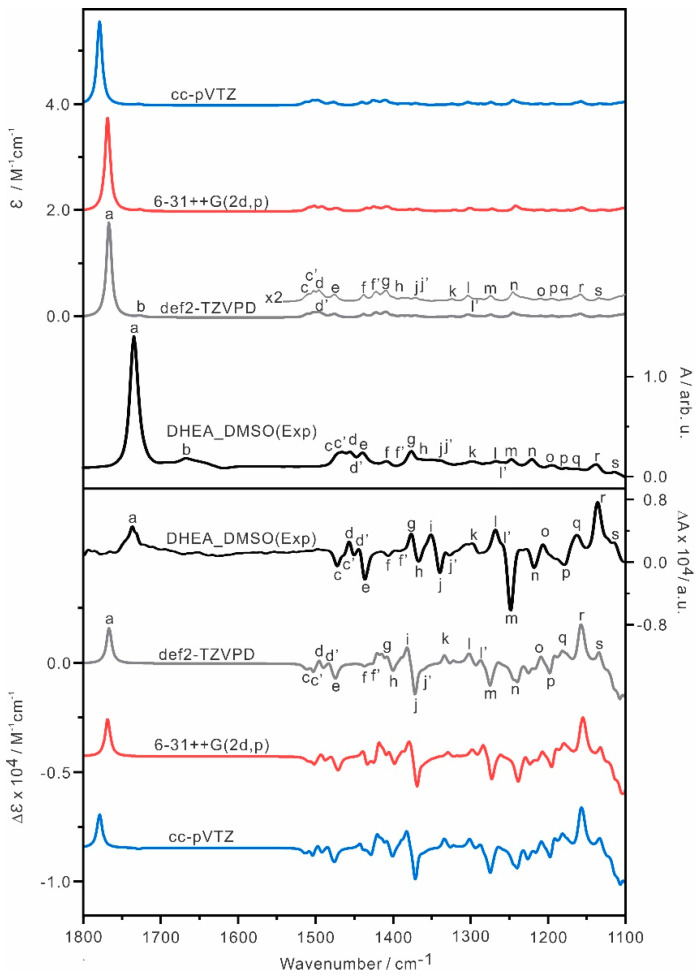
Comparisons of the experimental IR and VCD spectra of DHEA in DMSO−*d*_6_ at room temperature with the Boltzmann averaged IR and VCD spectra of the DHEA conformers with the B3LYP−D3BJ functional and three different basis sets with PCM of DMSO. The experimental units are given on the right side. The main IR and VCD band features are labelled as ‘a’ to ‘s’ to aid the comparison.

The Boltzmann averaged IR and VCD spectra of the three most stable conformers of DHEA were simulated using three different basis sets: cc-pVTZ, 6-31++G(2d,p) and def2-TZVPD. The B3LYP-D3BJ/def2-TZVP has been utilized extensively by the rotational spectroscopic community and the inclusion of an additional set of diffuse functions, i.e., def2-TZVPD, appeared to provide even better performance for conformational geometries and their energy ordering, with little additional computational cost [40]. It is interesting to compare its performance with the other two basis sets often used by the VCD and ROA community. The simulated spectra are compared with the experimental spectra in Figure 3, while the simulated IR and VCD spectra of individual DHEA conformers at the B3LYP-D3BJ/def2-TZVPD level are presented in Figure S5. The relative free energies and Boltzmann population factors of each conformer obtained with each basis set are compared in Table S1. Overall, the simulated spectra obtained with the three different basis sets are quite consistent with each other.

To assist the comparison between the experimental and theoretical simulations, the correspondence IR and VCD spectral features from high to low cm^−1^ are labelled alphabetically with ‘a’ to ‘s’. In general, the theoretical IR and VCD spectra capture the main experimental features well, indicating that the conformers identified are the dominant ones in DMSO. Overall, the B3LYP-D3BJ/def2-TZVPD level provides the best agreement with the experiment, for example, the carbonyl bands predicted are closest to the experimental wavenumbers among the three levels of theory used. We therefore use the B3LYP-D3BJ/def2-TZVPD level of theory in the reminder of the paper.

**Figure 4:**
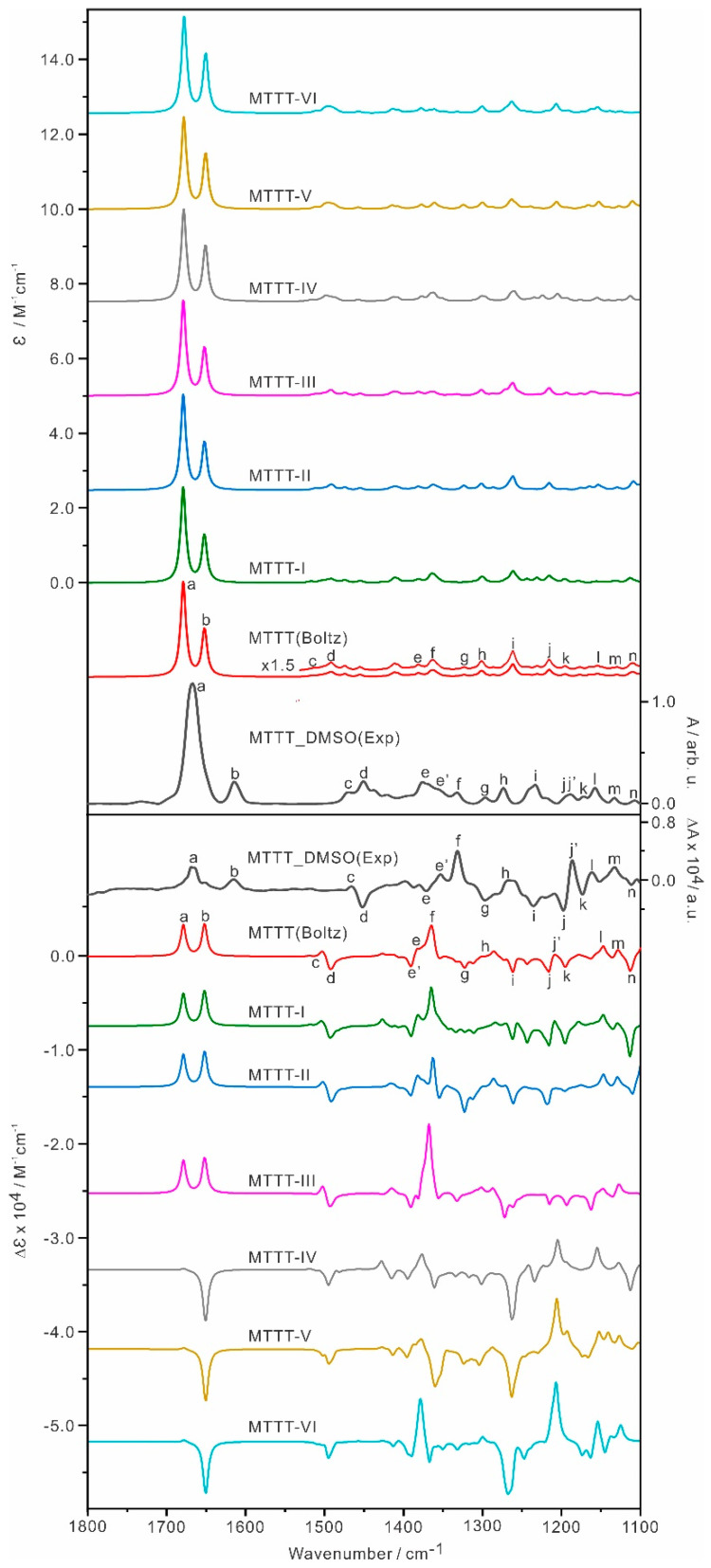
Simulated IR and VCD spectra of the six most stable MTTT conformers, MTTT−I to −VI and the related Boltzmann averaged IR and VCD spectra at the B3LYP−D3BJ/def2−TZVPD/PCM(DMSO) level of theory at 298 K. The experimental units are on the right side. The main IR and VCD band features are labelled as ‘a’ to ‘n’ to aid the comparison.

Next, the Boltzmann averaged IR and VCD spectra of MTTT and the corresponding individual conformer spectra are depicted in Figure 4. To guide the comparison, all visible features are also labelled as ‘a’ to ‘n’ in the spectra. In general, the Boltzmann average IR and VCD spectra exhibit a very good agreement with the experiment, and below we examine some specific features more closely. Looking at the contributions of each individual IR and VCD spectrum, MTTT-I, MTTT-II and MTTT-III show very similar IR and VCD spectral features. The main proof for the existence of MTTT-II and -III come from the bands labelled as ‘e’ to ‘i’, where some strong negative and positive VCD features are observed, but are less obvious in the VCD spectrum of MTTT-I.

How about the existence of the higher energy A ring conformations? Based on the simulated spectra, while the IR spectra of MTTT-IV to -VI exhibit similar spectral features to those of MTTT I-III, significant differences are present in the VCD spectra of these two groups of conformers. For example, a very weak positive and strongly negative VCD bands at C=O and C=C, i.e., ‘a’ and ‘b´, were predicted, respectively, for both MTTT-IV and V, whereas the corresponding VCD features of MTTT-I to -III are both positive with similar intensity. Experimentally, band b is weaker than band a in the VCD spectrum. This can be understood in terms of the contribution by MTTT-IV and V and it is likely that the contribution of IV and V is somewhat larger than the predicted Boltzmann factors, leading to a smaller intensity for band b than band a in the VCD spectrum, as observed experimentally.

**Figure 5:**
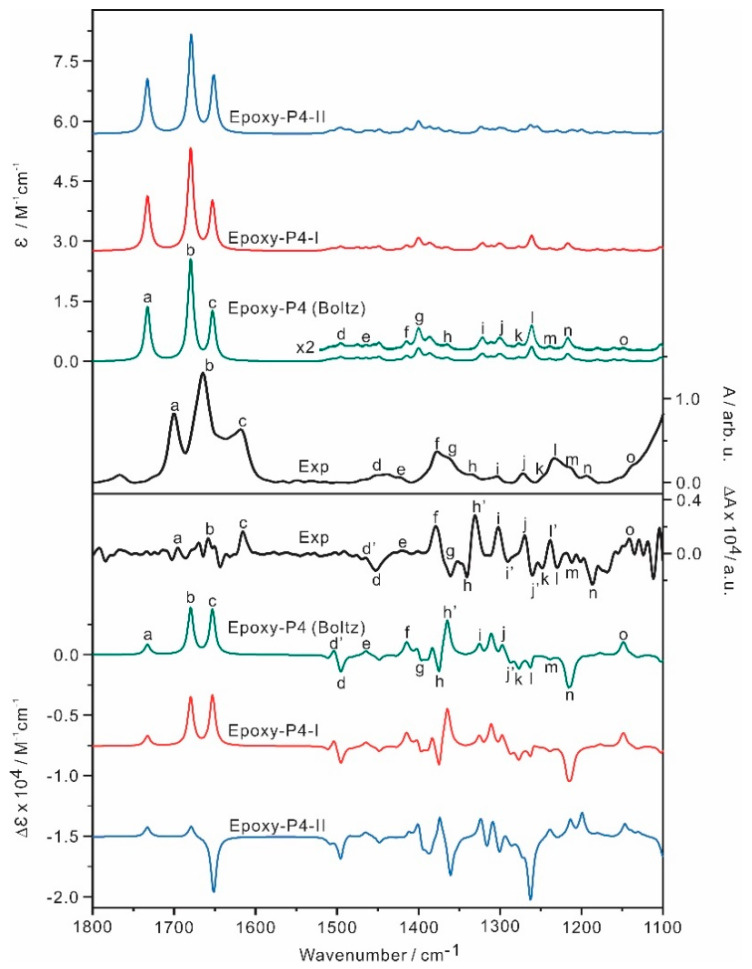
Simulated IR and VCD spectra of the two most stable Epoxy−P4 conformers, Epoxy−P4−I and −II, and the related Boltzmann averaged IR and VCD spectra at the B3LYP−D3BJ/def2−TZVPD/PCM(DMSO) level of theory at 298 K. The experimental units are on the right side. The main IR and VCD band features are labelled as ‘a’ to ‘o’ to aid the comparison.

For Epoxy-P4, the average and individual conformer IR and VCD spectra are compared with the experimental data in Figure 5. While the simulated IR spectra of Epoxy-P4-I and -II appear very similar, their VCD features are predicted to differ drastically. In the above 1600 cm^−1^ region, there are three main experimental IR bands, corresponding to the substituted acetyl C=O (ring D) stretch, the keto C=O (ring A) stretch, and the C=C stretch from high to low wavenumbers. The frequency ordering and their relative intensity are very well reproduced theoretically, although the relative position of band b with respect to band a and band c is not as well captured. There are also two medium intensity, somewhat broad experimental IR bands at ~1370 cm^−1^ and 1240 cm^−1^. Again, these are well captured by the averaged simulated IR spectrum. Similarly, the experimental VCD features in the below 1600 cm^−1^ region are well reproduced by the averaged VCD spectrum. All these evidences strongly indicate that Epoxy-P4-I is indeed the most dominant geometry in solution in the current experiment.

In the above 1600 cm^−1^ region, a positive VCD band, labelled as ‘c’, was observed for the C=C stretch, similar to that in the related MTTT, and was correctly predicted. In addition, a tiny positive VCD band, labelled as ‘a’, was predicted for the acetyl C=O band. The corresponding experimental VCD feature for ‘a’ was tentatively assigned based on its experimental IR band position. The positive VCD feature of the keto C=O (ring A) stretch predicted, labelled as ‘b’, was difficult to identify in the experimental spectrum. Since the VCD features of Epoxy-P4-I and -II, which differ in their ring A conformations, are quite dissimilar, we also looked into the possibility that the rotation of the acetyl C=O group might alter the appearance of VCD noticeably. Therefore, a one-dimensional relaxed PES scan was carried out along the dihedral angle (C13-C8-C19-C23) in Epoxy-P4-I (Figure S6). The simulated IR and VCD spectra at some selected dihedral angles are shown in Figure S7. As one can see, there is little change in the IR features, whereas the sign of the VCD band of the acetyl C=O group flips and many other changes are also noted in the 1250–1450 cm^−1^ region. Interestingly, there is little change in the predicted VCD feature of the keto C=O stretch, indicating that consideration of this potential large amplitude motion in solution does not explain the somewhat worse agreement for the VCD band of the keto C=O stretch. One may speculate that this less satisfactory agreement may be related to the inadequate DFT performance to reproduce the resonance effect between C=C and keto C=O. This aspect will be further explored in the case of AcO-DHEA in Section 2.5.

### 2.5. Experimental and Simulated IR and VCD Spectra of AcO-DHEA

**Figure 6:**
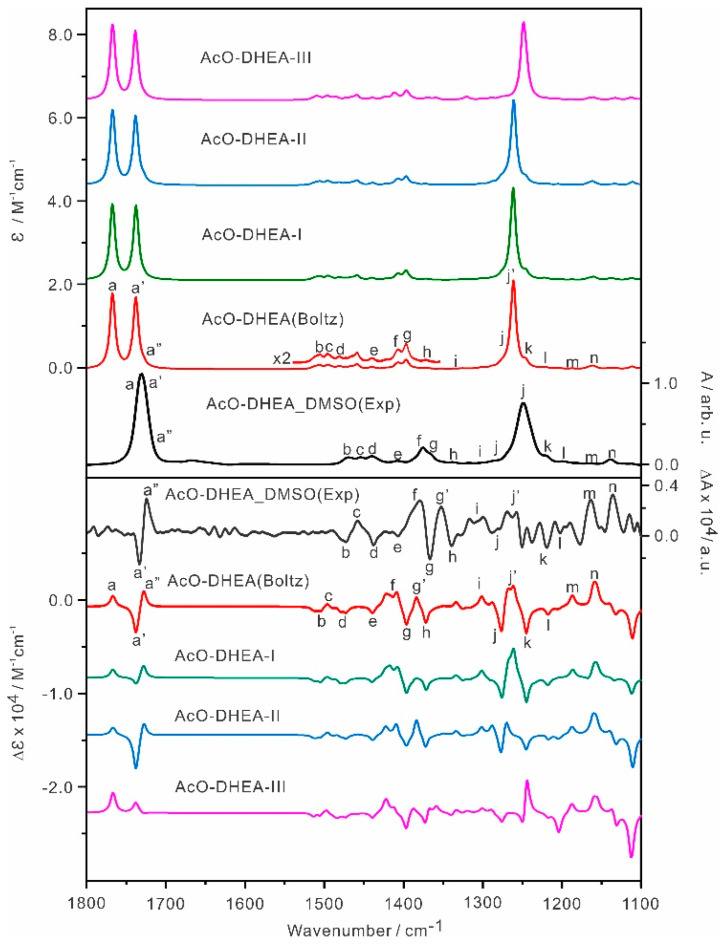
Simulated IR and VCD spectra of the three most stable AcO−DHEA conformers, AcO−DHEA−I to −III, and the related Boltzmann averaged IR and VCD spectra at the B3LYP−D3BJ/def2−TZVPD/PCM(DMSO) level of theory at 298 K. The experimental units are on the right side. The main IR and VCD band features are labelled as ‘a’ to ‘n’ to aid the comparison.

For AcO-DHEA, the individual and Boltzmann averaged IR and VCD spectra of AcO-DHEA-I to III are shown in Figure 6. To assist the comparison, all the corresponding visible bands are labelled alphabetically as ‘a’ to ‘n’. Overall, the simulated IR and VCD spectral features show a reasonably good agreement with the experimental data. One exception is the bands in the 1700–1800 cm^−1^ region. There are three vibrational bands predicted in this region: the C=O stretch of cyclopentanone, band a, the C=O stretch of AcO, band a′, and the C=C stretch, band a′′ going from high to low cm^−1^. In the IR prediction, the C=C band (band a″) is very weak and buried in the lower wavenumber side of the C=O band (band a´), whereas in the corresponding VCD features, we have a negative band a′ and a positive band a′′ with medium intensity. One can correlate the experimental VCD −/+ features to the predicted −/+ features of band a′ and band a′′. How about the C=O stretch of cyclopentanone, i.e., band a? It is obvious that the gap between band a and band a′ was predicted to be much larger than that observed experimentally. In fact, in the experiment, the two C=O bands merge into one slightly broader band. Furthermore, if one moves band a closer to band a′ as implied by the experimental IR feature in this region, the positive VCD band a would cancel out some negative VCD intensity of band a′, leading to better relative intensity agreement with the experimental −/+ VCD features. Overall, one can conclude that the DFT calculation at this level of theory accurately captures the IR and VCD features of AcO-DHEA, except for the narrow gap between the C=O stretch of cyclopentanone and C=O of the AcO group.

Below we explore several potential sources which may account for the IR frequency discrepancy in the >1700 cm^−1^ region discussed above: (1) solvent effects; (2) possible large amplitude motions in solution; (3) different levels of theory, specifically different functionals.

**Figure 7:**
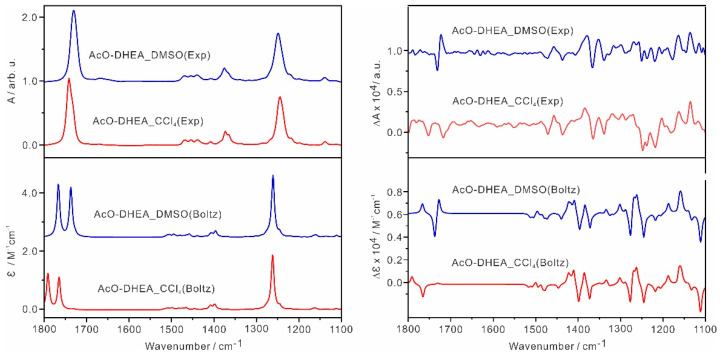
Comparison of the experimental IR and VCD spectra of AcO−DHEA in DMSO (blue) and in CCl_4_ (red) and the corresponding Boltzmann averaged spectra at the B3LYP−D3BJ/def2−TZVPD level of theory with the inclusion of PCM of DMSO and CCl_4_ at 298 K.

First, we examined whether the DMSO solvent causes these bands (>1700 cm^−1^) to merge into one or not. The IR and VCD spectra of AcO-DHEA were subsequently measured in CCl_4_, and their experimental spectra are compared with those obtained in DMSO in Figure 7. It is clearly that these two sets of experimental data exhibit much similarity in both IR and VCD features, including the fact that only one IR band (>1700 cm^−1^) was observed in CCl_4_, similar to that in DMSO. We can safely say that solvents do not cause these predicted split bands to merge together. Some noticeable differences in these solvents are: (1) the broad carbonyl band, which is located at ~1731 cm^−1^ in DMSO-*d*_6_, is blue shifted by ~11 cm^−1^ in CCl_4_; (2) the associated VCD features also change from −/+ in DMSO to −/− in CCl_4_; and (3) the IR C-O band around 1250 cm^−1^ is slightly red shifted from DMSO to CCl_4_, opposite to that of the carbonyl band.

We further evaluated if the current DFT calculations can provide a satisfactory explanation of the observed changes with DMSO and CCl_4_ described above. The main difference between DMSO and CCl_4_ is their dielectric constants: ε = 46.826 for DMSO and ε = 2.228 for CCl_4_. The relative free energies and Boltzmann population factors of the most three stable AcO-DHEA conformers in DMSO and CCl_4_ are compared in Table S2. Interestingly, the relative energy ordering of AcO-DHEA-I and AcO-DHEA-II flips from DMSO to CCl_4_, whereas AcO-DHEA-III remains much higher in energy with a Boltzmann factor of less than 0.5%. Please note that we keep the -I to -III labels the same as in DMSO to avoid confusion.

The Boltzmann averaged IR and VCD spectra DMSO and CCl_4_ are compared in Figure 7 together with the experimental data, while the individual conformer IR and VCD spectra in CCl_4_ are given in Figure S8. Indeed, the experimental blue shift and the smaller red shift described above were captured by these DFT calculations. In terms of VCD features, the simulated pattern remains essentially the same in the below 1600 cm^−1^ range. In the above 1700 cm^−1^ range, the “+/−/+” prediction from high to low cm^−1^ in DMSO is replaced by “+/−/+(very weak)” in CCl_4_. One can tentatively assign the low experimental ‘-/no visible feature’ in CCl_4_ to the simulated “−/+(very weak)” in CCl_4_. The main discrepancy between experiment and theory is again associated with the acetyl C=O stretch.

Next, we consider the effects of potential large amplitude motions of AcO-DHEA in solution. It has been recognized from some previous ab initio MD studies [28] and DFT VCD solvation studies [66] that using the spectroscopic properties at the predicted minima may not be enough to reproduce the observed spectral features in solution. More recently, some rotational spectroscopic studies [40,67] demonstrated clearly that the experimental electric dipole components and nuclear quadruple coupling constants could be very different from those predicted at the minima because of large amplitude motions. In addition, the associated VCD simulations showed that the vibrationally average features can be dramatically different from those at the minima [40]. Following a similar approach described in [66], we first performed a one-dimensional, relaxed potential energy surface (PES) along the dihedral angle (C19-C21-O2-C49) for 360° with the PCM of DMSO and the result is depicted in Figure S9. The low energy conformers, AcO-DHEA-I, II and III can all be identified along this scan path. The energy barrier separating AcO-DHEA-I and AcO-DHEA-II is quite low (<2.5 kJ mol^−1^). One may expect the compound to undergo a large amplitude motion between I and II in solution and the resulting IR and VCD spectra would have contributions from all geometries in between [40]. In contrast, AcO-DEHA-III is separated from AcO-DHEA-II and -I by somewhat high barriers of ~20 kJ mol^−1^.

To visualize the effects of rotating this specific dihedral angle on the IR and VCD spectra, a few geometries near the minima and the transition states are selected for spectral simulations. The resulting IR and VCD spectra at each angle are depicted in Figure S10. As one scans through dihedral angle, the only visible change in IR is associated with the C-O bond of AcO, whereas the three bands in the above 1700 cm^−1^ region hardly change. The C-O stretching band of AcO experiences a noticeable red shift around the transition states from its values at AcO-DHEA-I and -II which are around 1250 cm^−1^. This might be caused by the tension C-O experiences as one rotates the dihedral angle (C19-C21-O2-C49). In terms of the simulated VCD features, the positive VCD sign of the pentanone C=O remains positive throughout, whereas the C=O of AcO not only undergoes much intensity change but also sign changes, so does the C=C stretch. These observations suggest that the VCD features around 1700 cm^−1^ might be difficult to capture by applying the usual DFT simulations at the minima, since other conformations further away from the minima might contribute noticeably to the current solution experiment. On the other hand, it is also obvious that rotation of this dihedral angle alone cannot bring the large predicted gap between band a and band a´ into agreement with experiment.

**Figure 8:**
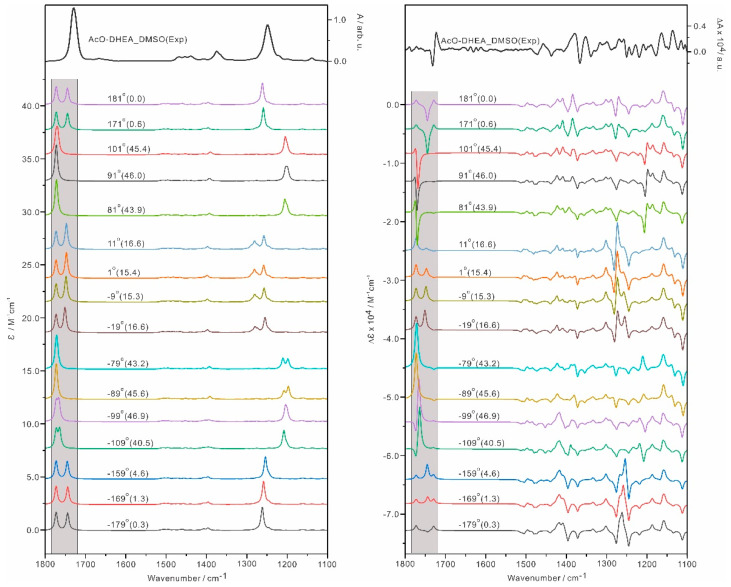
Simulated IR and VCD spectra of AcO−DHEA at several dihedral angle (C21−O2−C49−C51) values along the PES (Figure S11) at the B3LYP−D3BJ/def2−TZVPD/PCM(DMSO) level at 298 K. The relative energies in kJ mol^−1^ are listed in the brackets.

A second one-dimensional relaxed PES scan was performed along the dihedral angle (C21-O2-C49-C51) in AcO-DHEA-I. The scan result is summarized in Figure S11. We note that the PES scan is not smooth, and it does not return to the same conformer after a full scan of 360°. This is not surprising because the geometry may slip into a new conformer which differs in another coordinate as discussed before [68]. The simulated IR and VCD spectra at several selected angles are given in Figure 8, exhibiting drastic changes along this dihedral angle. For the conformations near the transition states, the AcO C=O frequency shifts noticeably to higher wavenumber and merges with that of the pentanone C=O band, whereas the very strong AcO C-O band shifts from ~1260 cm^−1^ much further to the lower wavenumber of 1200 cm^−1^. At the transition states, the C=O and C-O groups of AcO are significantly twisted with respect to each other, therefore removing their “conjugation” and shifting the C=O band further to the blue and the C-O band further to the red. Not surprisingly, large differences are also observed in their corresponding VCD spectra in the two regions discussed above. However, no particular dihedral conformations offer a better agreement to the experimental IR and VCD spectra than the above prediction based on the minima. In summary, consideration of the flexibility of the AcO group will likely alter the IR and VCD appearance in the >1700 cm^−1^ region, although none of the geometries considered offer a better agreement between experiment and theory.

The corresponding simulations at different dihedral angle values were carried out for AcO-DHEA in CCl_4_ and a similar conclusion could be drawn. For conciseness, we put the IR and VCD simulations of AcO-DHEA in CCl_4_ and the additional discussions on the comparison between experiment and theory into Point S1.

Recently, the performance of some hybrid and double-hybrid functionals in conjunction with partially augmented double- and triple-zeta basis sets in predicting IR band frequency and intensity has been evaluated [69] where the double-hybrid functional B2PLYP shows excellent performance. In addition, several hybrid functionals were tested to reproduce the IR spectra of the open and closed isomers of four diarylethenes, and the authors recommended to use the dispersion-corrected PBE0 functional [70]. Below we tested the performance of PBE0 [71]-D3BJ/def2-TZVPD and a mixed single and double-hybrid functional method [72] (vide infra) for the IR and VCD spectra of AcO-DHEA.

**Figure 9:**
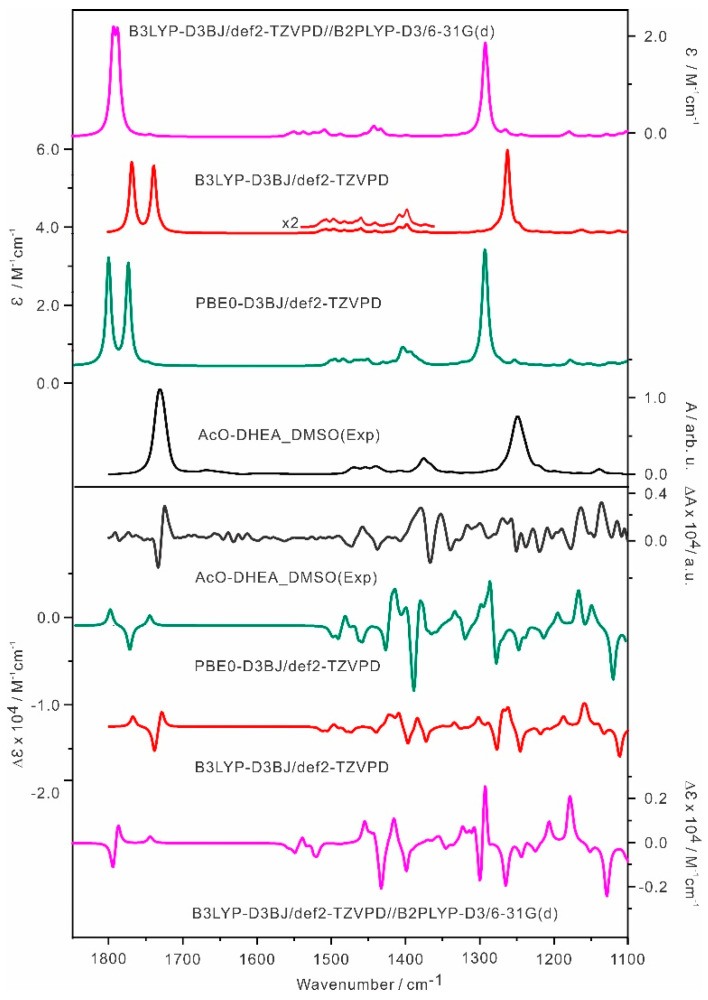
Comparison of the experimental and simulated IR and VCD spectra of AcO−DHEA at these different levels: B3LYP−D3BJ/def2−TZVPD, PEB0−D3BJ/def2−TZVPD and B3LYP−D3BJ/def2−TZVPD//B2PLYP−D3/6−31G(d) with the PCM of DMSO.

The averaged IR and VCD of AcO-DHEA at the PBE0-D3BJ/def2-TZVPD level are summarized in Figure 9 and compared with the experiment and those simulated with B3LYP-D3BJ. While the PBE0 simulations do not solve the issue associated with the IR band frequencies in the >1700 cm^−1^ region, the IR and VCD patterns in the <1600 cm^−1^ region appear to agree even better with the experimental ones than those predicted with the B3LYP-D3BJ functional if one shifts the whole spectrum to a lower wavenumber. More specifically, the IR band shapes in the 1300–1500 cm^−1^ region and their relative intensity to the strong band at 1250 cm^−1^ were also better captured at the PBE0 level. So were the detailed VCD features in the same region.

Next, we applied the double-hybrid B2PLYP functional at the B2PLYP-D3/6-31G(d) level. Attempts to use larger basis sets, such as 6-311+G(2d,p) were not successful because of the significant demand on the computational resource. In addition, it is currently not yet possible to calculate VCD using directly the double-hybrid functionals. We therefore utilized the ‘mixed’ method available in the development version of Gaussian (GDV) [71]. In this method, for example, the B3LYP-D3BJ/def2-TZVPD calculations of the atomic polar tensors (APTs) and atomic axial tensors (AATs), using the double-hybrid B2PLYP-D3/6-31G(d) optimized geometry and force field, were computed using a variational response-function formulation as suggested by Coriani et al. [73]. We denote this ‘mixed’ method as B3LYP-D3BJ/def2-TZVPD//B2PLYP-D3/6-31G(d). The implementation includes magnetic field dependent basis functions (GIAOs) which ensure gauge origin-independent results. In this approach, the APTs and AATs are computed at the DFT level and then combined with the double-hybrid normal modes. The results of the mixed method at the B3LYP-D3BJ/def2-TZVPD//B2PLYP-D3/6-31G(d) level are summarized in Figure 9, while the related individual IR and VCD of AcO-DHEA-I and -II are depicted in Figure S15. As one can see, the mixed method with the double-hybrid functional improves the overall agreement with the experimental IR and VCD spectra, especially the IR band spacing in the >1700 cm^−1^ region and the associated VCD features. For completeness, the ‘mixed’ results with the PCM of CCl_4_ are also summarized in Figure S14 of Point S1 where the double-hybrid functional improves the overall agreement with the experimental IR spectra. In the >1700 cm^−1^ region, the blue shifts of the C=O bands from DMSO to CCl_4_ were reproduced, although the associated VCD sign was predicted to be ‘−/+’ not in total agreement with the experimental ‘−/−’. Overall, it is very encouraging, emphasizing the role of the double-hybrid functional in predicting the accurate IR frequency pattern.

In Figure S16, we compare the simulated IR spectra of AcO-DHEA-I and -II at the mixed B3LYP-D3BJ/def2-TZVPD//B2PLYP-D3/6-31G(d) level versus at the B2PLYP-D3/6-31G(d) level. Almost the same IR spectra were obtained at these two levels, except for the IR intensity at the C=O stretching region which is a bit higher with the mixed method in comparison with the pure double-hybrid method. To evaluate whether a much smaller basis set at the B3LYP level would affect the performance of the mixed method, we compare the simulated IR and VCD of AcO-DHEA-I at the B3LYP-D3BJ/6-311+G(2d,p)//B2PLYP-D3/6-31G(d) and at the B3LYP-D3BJ/6-31G(d)//B2PLYP-D3/6-31G(d) level in Figure S17. The results are almost identical at these two levels, suggesting that the size of the basis sets plays only a minor role here.

Please note that the energy ordering remains the same for AcO-DHEA-I and -II with the ‘mixed’ and the B3LYP method, whereas the obtained geometries differ only slightly. For example, the dihedral angle (C19-C21-O2-C49) values with the PCM of DMSO for AcO-DHEA-I are −153.05° and −155.35° and for AcO-DHEA-II are −85.13° and −82.93° with the B3LYP and the ‘mixed’ method, respectively. The sign change for the ring C=O and Ac C=O modes between the B3LYP//B2PLYP and the usual B3LYP calculations are related to the evaluations of AATs and APTs which depend on the geometries and normal modes. The AATs and APTs are related to the electric dipole (E) and magnetic dipole (M) transition moments for each mode where the rotational strength is Ex × Mx + Ey × My + Ez × Mz. For example, for the ring C=O mode at the B3LYP//B2PLYP level with the PCM of DMSO, we have Ex × Mx = 140, Ey × My = 569 and Ez × Mz = −731 so that the sum is −21 in units of 10^−44^ esu^2^ cm^2^. At the B3LYP level, we have Ex × Mx = 553, Ey × My = −678, Ez × Mz = 139 and the sum is +14 in units of 10^−44^ esu^2^ cm^2^. So, the combination of the slightly different geometry and quite different normal modes produces different electric dipole and magnetic dipole transition moment components which in turn lead to differing signs and magnitudes of the rotational strengths. Generally, the double-hybrids, such as B2PLYP, are expected to produce better normal modes, which in the case of AcO-DHEA, seems to matter.

### 2.6. Brief Comments on the Absolute Configurations of the Four Steroids

From the conformational analyses for each steroid hormone system, it is clear that one can still confidently investigate the conformational distributions based on the DFT simulations at the minima identified. The stereogenic centers used for the IR and VCD simulations are 8R, 9S, 10R, 13S, 14S and 17S for MTTT, 3S, 8R, 9S, 10R, 13S and 14S for DHEA, 3S, 8R, 9S, 10R, 13S and 14S for AcO-DHEA, and 8R, 9S, 10R, 13S, 14S, 16R and 17S for Epoxy-P4, which are also labelled in Scheme 1. Alternating chirality of some or all of these stereogenic centers would in principle generate some different diastereomers, i.e., different steroid systems, or the opposite enantiomers, respectively. It is clear that the simulated IR and VCD spectra would be highly sensitive to all these modifications. The good agreements between the simulated and the experimental data of the four steroids have been generally obtained for all four steroids, allowing one to confirm their stereochemistry labels and also their conformational distributions. The discrepancies detected in the carbonyl stretch region indicate that further theoretical improvements are needed to capture the relative frequencies more accurately in this region.

## 3. Materials and Methods

### 3.1. Experimental

MTTT, DHEA, AcO-DHEA and Epoxy-P4 (HPLC purity > 98%) were purchased from CHENG-DU MUST BIO-TECHNOLOGY CO., LTD., (Chengdu, China) and used without further purification. DMSO-*d*_6_ and CCl_4_ were purchased from Sigma Aldrich (St. Louis, MA, USA). The IR and VCD spectra were measured using a FTIR spectrometer (Bruker Vertex 70), equipped with a VCD module (PMA 50) [74]. The photoelastic modulator (PEM) was set at 1400 cm^−1^ for all measurements. The signal detection occurred at the liquid nitrogen cooled mercury cadmium telluride (MCT) detector. The total acquisition time was 3 h (3 × 1 h) or about 13,000 scans with 4 cm^−1^ resolutions. The spectra were recorded in the region of 1100–1800 cm^−1^. MTTT and DHEA were measured in DMSO-*d*_6_ at a concentration of 80 mg/mL. AcO-DHEA and Epoxy-P4 were measured in DMSO-*d*_6_ at a concentration of 25 mg/mL and the same concentration was used for AcO-DHEA in CCl_4_. A 0.1 mm spacer was employed for all measurements. The concentration and the path length were optimized to get the IR absorbance in the range of 0.2–0.9, in order to obtain reproducible VCD spectra. The reported IR and VCD spectra were baseline corrected by the subtraction of the solvent spectrum, measured under identical conditions.

### 3.2. Theoretical

The CREST code [43] was utilized to systematically explore the possible conformers of MTTT, DHEA, AcO-DHEA and Epoxy-P4 with the inclusion of the generalized Born (GB) based GBSA implicit solvation model, using dimethyl sulfoxide (DMSO) or CCl_4_ as the solvent [75]. The CREST code is built upon the previous semiempirical tight-binding (TB) quantum chemistry method by Grimme and co-workers, called GFN-xTB [76]. The new CREST code is designed for fast and reliable exploration and screening of the conformational space of mid- to large-sized molecules with up to about a thousand atoms. It can automatically perform multiple MD runs for conformational exploration and has a built-in feature to compare and discard redundant geometries.

The further conformational search, geometry optimization and harmonic frequencies calculations were performed through a multi-tiered approach developed before [47] which include several steps: (1) search for all the CREST candidates; (2) the DFT optimizations of the CREST candidates with a relaxed convergence criterion at the revPBE-D3/def2-SVP [51] level, with the empirical D3 dispersion correction; (3) a single-point energy evaluation at the B3LYP-D3/def2-TZVP level of the optimized structures in step (2). The steps (2) and (3) were performed using Molpro [77]; (4) The final geometry optimization and harmonic frequency IR and VCD calculations were performed using the Gaussian 16 package [78]. All calculations were done at the B3LYP-D3BJ/def2-TZVPD level. The implicit solvent was included using the integral equation formalism (IEF) version of the PCM to account for the bulk solvent environment (ε = 46.826 for DMSO and ε = 2.2280 for CCl_4_). Some additional calculations were also carried out at the B3LYP/cc-pVTZ, B3LYP/6-31++G(2d,p), and PEB0-D3BJ/def2-TZVPD levels, as well as the mixed calculations at the B3LYP-D3BJ/def2-TZVPD//B2PLYP-D3/6-31G(d), B3LYP-D3BJ/6-311+G(2d,p)//B2PLYP-D3/6-31G(d) and B3LYP-D3BJ/6-31G(d)//B2PLYP-D3/6-31G(g) levels using the development version of Gaussian [71]. A Lorentzian line shape with a half-width at half-height (HWHH) of 4 cm^−1^ was used for the simulation of IR and VCD spectra. No frequency scaling factors were used.

## 4. Conclusions

The stereochemical information and especially the conformational distributions of the four steroid hormones, DHEA, MTTT, AcO-DHEA and Epoxy-P4, were investigated using a combined experimental and theoretical approach with IR and VCD spectroscopy. While these four steroids share some structural similarity, their experimental IR and VCD spectra are distinct from each other, allowing one to distinguish them individually. Indeed, we were able to utilize the IR features to correctly identify one sample which was initially mislabeled. Systematic conformation searches by CREST were carried out to identify the lower energy conformers which were used for further IR and VCD simulations. In general, good agreements between the experimental and simulation IR and VCD spectra at the B3LYP-D3BJ/def2-TZVPD and PBE0-D3BJ/def2-TZVPD levels were achieved, confirming that the dominant conformations predicted are those observed in solution. Some noticeable disagreements are found in the above 1700 cm^−1^ region for the IR and VCD spectra of AcO-DHEA. Specifically, the theoretical calculations with the single hybrid functionals all predicted two well separated band positions for the C=O stretching bands of acetyl and cyclopentanone, whereas the experimental IR spectra in DMSO show only one slightly broadened band. The possible causes of the disagreement, such as solvent effects, large amplitude motions, and some deficiency of the DFT calculations, were carefully examined. The related experimental and theoretical IR and VCD spectroscopic study with the CCl_4_ solvent confirmed that solvent-solute interactions were not responsible for the disagreement, neither were the potential large amplitude motions in AcO-DHEA. A further focus is on the ´mixed´ calculations at the B3LYP-D3BJ/def2-TZVPD//B2PLYP-D3/6-31G(g) level with the double-hybrid functional. The use of the double-hybrid functional produced the IR frequency pattern in the whole 1100–1800 cm^−1^ region in agreement with the experiment, especially in the >1700 cm^−1^ region. The current work showcases the power of IR and VCD spectroscopy, aided by theoretical calculations, in providing structural information including conformational distributions of steroids in solution.

## Data Availability

Data is contained within the article.

## Supplementary Materials

The following supporting information can be downloaded at: https://www.mdpi.com/article/10.3390/molecules28020771/s1, Scheme S1: Molecular formula of some common steroids; Tables S1 and S2: Relative energies of the conformers of DHEA and AcO-DHEA; Figure S1: Comparison of IR spectra of two steroid samples with other known steroids and the theoretical IR spectra of MTTT-I and DHEA-I; Figures S2 and S3: Experimental IR and VCD spectra of the initial sample labelled as ‘MTTT’ in CCl_4_ and the related GC-MS results; Figure S4: Optimized geometries of MTTT-I and -IV; Figures S5, S7, S8 and S10: Simulated IR and VCD spectra of several conformers of DHEA, Epoxy-P4, and AcO-DHEA; Figures S6, S9 and S11: PES scans of Epoxy-P4 and AcO-DHEA; Point S1 and Figures S12–S14: Comparison of the simulated and experimental AcO-DHEA in CCl_4_; Figures S15–S17: Comparison of the ‘mixed’ and regular method for IR and VCD spectra of AcO-DHEA.
