# Supplementary material for: Conformations of Steroid Hormones: Infrared and Vibrational Circular Dichroism Spectroscopy
Source: Molecules. 2023 Jan 12;28(2):771. doi: 10.3390/molecules28020771 (PMC9864676; doi:10.3390/molecules28020771)
Supplement: Supplementary file 1 [file molecules-28-00771-s001.zip › molecules-2152520-supplementary.pdf]

molecules

MDPI

Supplementary Material

Conformations of Steroid Hormones: Infrared and Vibrational Circular Dichroism Spectroscopy

Yanqing Yang [1], Anna Krin [1,2], Xiaoli Cai [1,3], Mohammad Reza Poopari [1], Yuefei Zhang [3], James R. Cheeseman [4] and Yunjie Xu [1,*]

1 Department of Chemistry, University of Alberta, Edmonton, AB T6G 2G2, Canada
2 Centre for Science and Peace Research (ZNF), Universität Hamburg, Bogenallee 11, 20144 Hamburg, Germany
3 Key Laboratory for Green Chemical Process of Ministry of Education, Wuhan Institute of Technology, Wuhan 430073, China
4 Gaussian Inc., 340 Quinnipiac St., Bldg., 40, Wallingford, CT 06492-4050, USA
* Correspondence: yunjie.xu@ualberta.ca; Tel.: +1-780-402-1244

Contents

Scheme S1. Comparison of some major steroids……………………………………………………………………………2

Figure S1. Comparison of IR spectra of two steroid samples with other known steroids 2

Figure S2-S3. Experimental IR and VCD spectra of ´MTTT´ in CCl_4 and the related GC-MS results 3

Figure S4. Optimized geometries of MTTT-I and -IV..4

Figure S5. Simulated IR and VCD spectra of several conformers of DHEA 5

Table S1. Relative free energies and Boltzmann factors of DHEA conformers 5

Figure S6-S7. Simulated IR and VCD spectra of several conformers of Epoxy-P4 6

Table S2. Relative free energies and Boltzmann factors of AcO-DHEA conformers..7

Figure S8. Simulated IR and VCD spectra of several conformers of AcO-DHEA...7

Figure S9. One PES scan of AcO-DHEA..8

Figure S10. Simulated IR and VCD spectra of AcO-DHEA at selected dihedral angle values...8

Figure S11. Second PES scan of AcO-DHEA 9

Point S1 Figure S12-14 Comparison of the simulated and experimental AcO-DHEA in CCl4.......................................9-12

Figure S15-17. Comparison of the 'mixed' and regualr method for IR and VCD spectra of AcO-DHEA....................13-14

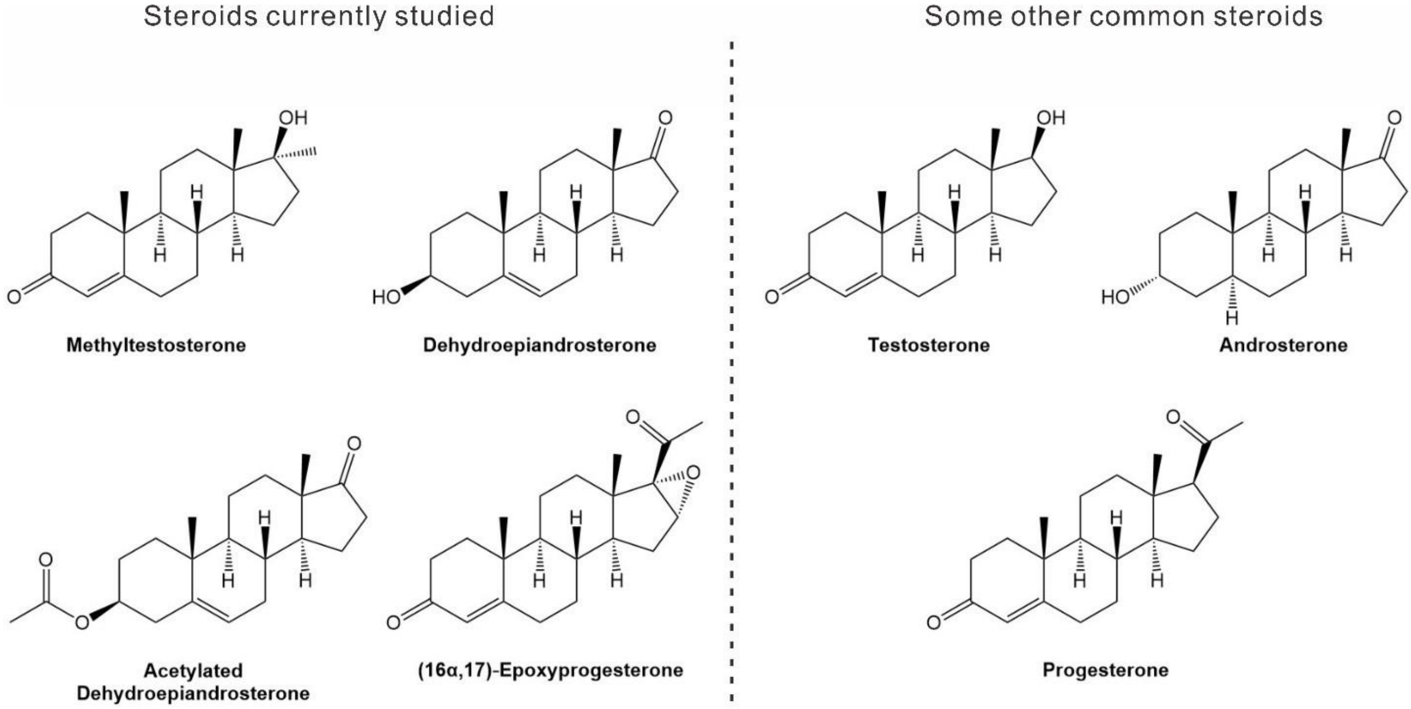

Scheme S1. The four steroid compounds studied in this paper and some other common steroids.

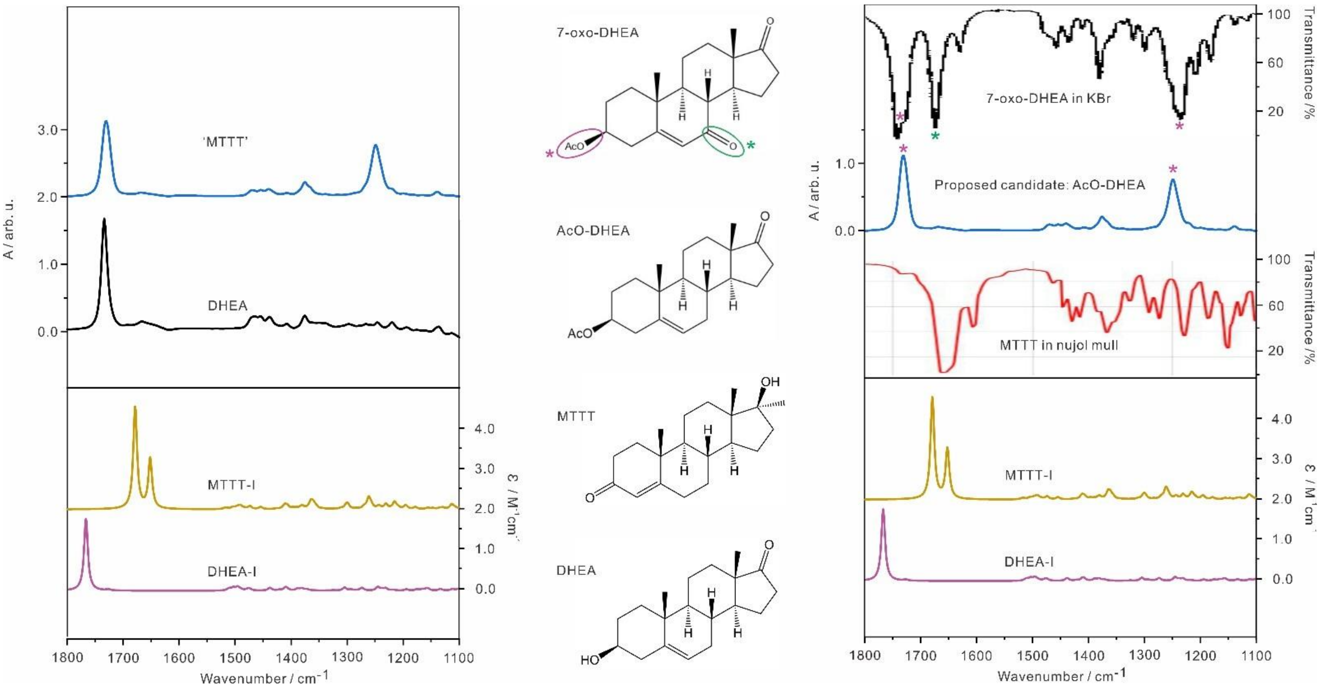

Figure S1. Left: Comparison of the experimental IR spectra (top) of the samples labelled as ´MTTT´ and ´DHEA´ in DMSO-d6 at room temperature with the simulated IR spectra (bottom) of the most stable conformer of MTTT and DHEA at the B3LYP-D3BJ/def2-TZVPD/PCM(DMSO) level of theory. Right: Comparison of the experimental IR spectra of the sample labelled as ´MTTT´ with the experimental IR spectra of 7-oxo-DHEA in KBr from Ref. 44 and MTTT (nujol mull) reported on the National Institute of Standards and Technology (NIST) website, i.e., Ref. 43. Center: the molecular formula of the four related compounds.

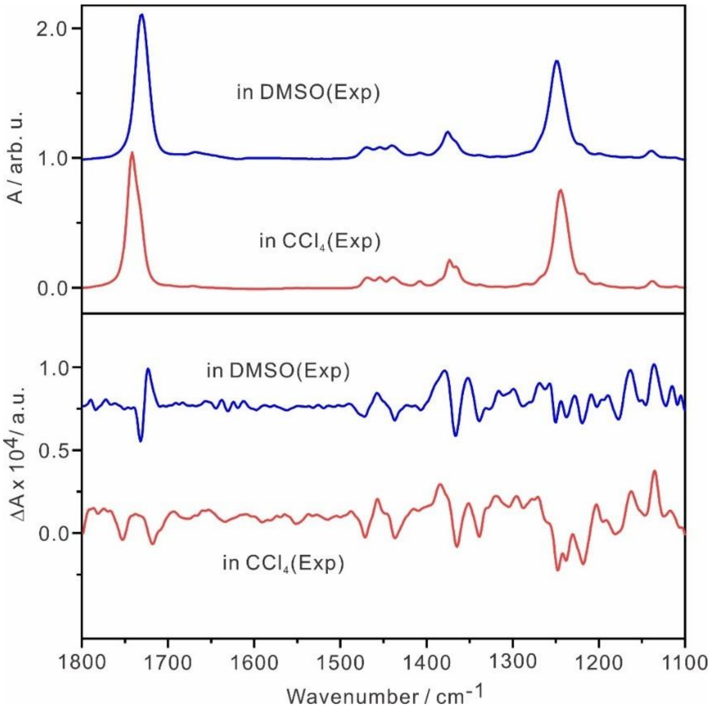

Figure S2. Experimental IR and VCD spectra of the initial sample labelled as ´MTTT´ in DMSO-d6 (blue) and in CCl_4 (red) at room temperature. This was latter correctly identified as AcO-DHEA.

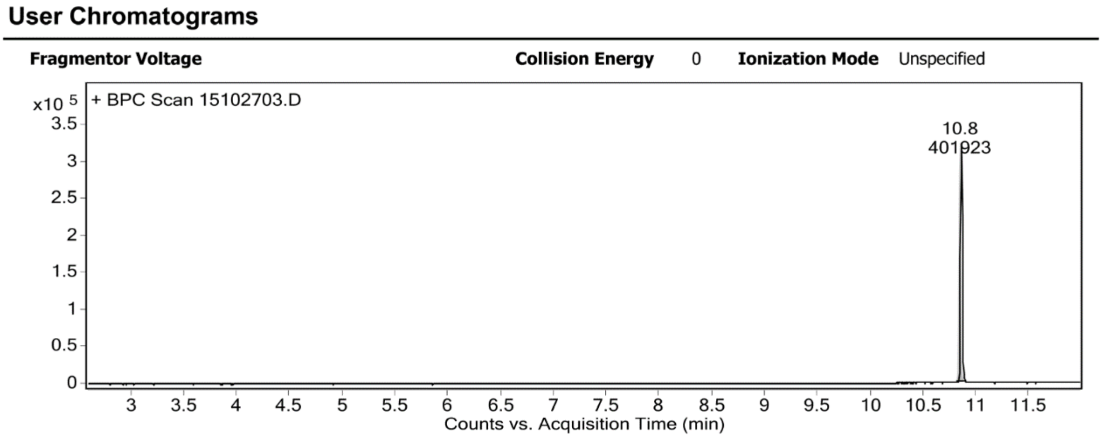

Integration Peak List

Peak	Start	RT	End	Height	Area	Area %
1	10.8	10.8	10.9	322105	401923	100

User Spectra

Figure S3. The GC-MS result of the initial sample labelled as ´MTTT´.

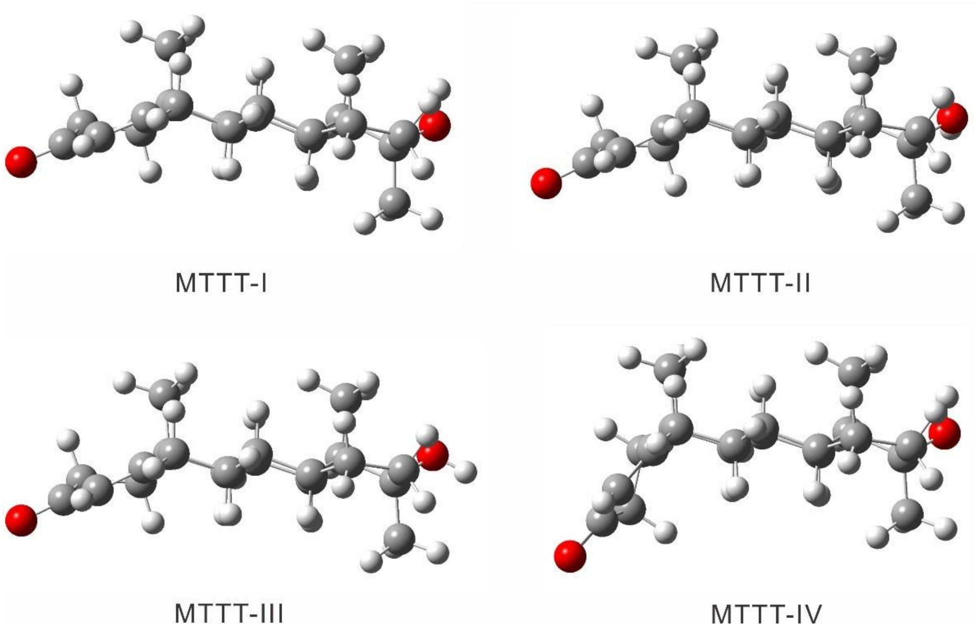

Figure S4. The optimized geometries of MTTT-I to MTTT-IV at the B3LYP-D3BJ/def2-TZVPD level of theory with the PCM of DMSO. The conformers are oriented in such a way so that one can see ring A (cyclohexene ring) takes on two different conformations in MTTT-I to -III versus in MTTT-IV.

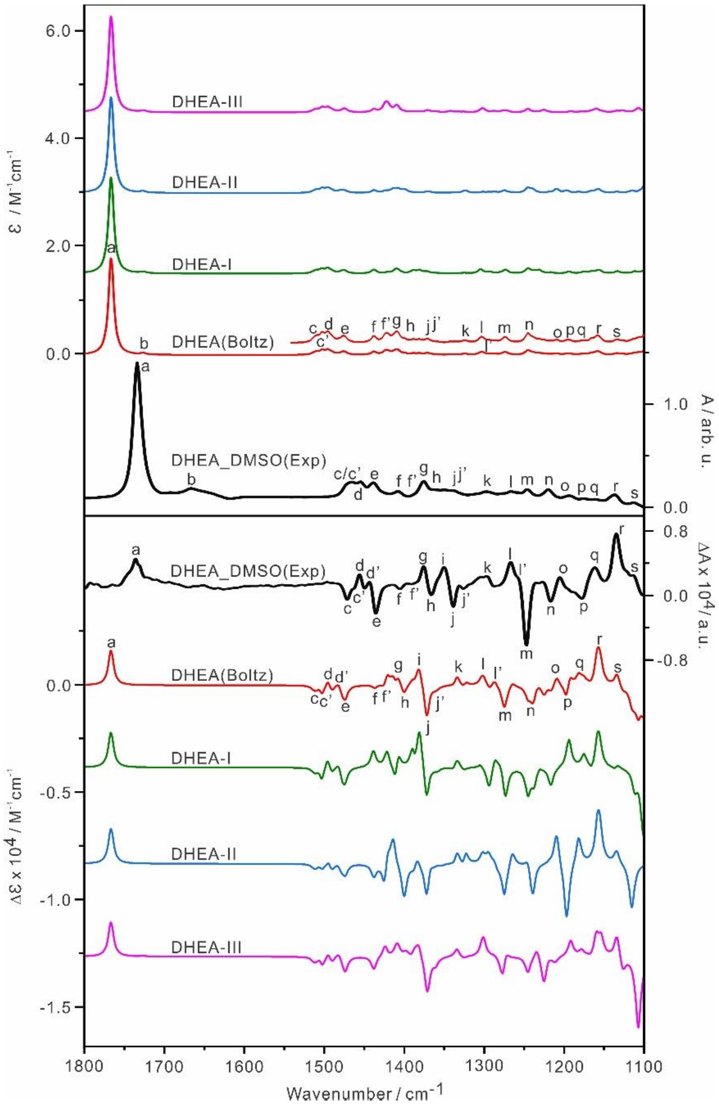

Figure S5. Simulated IR and VCD spectra of the three most stable DHEA conformers, DHEA-I-III and the related. Boltzmann averaged IR and VCD spectra at the B3LYP-D3BJ/def2-TZVPD/PCM(DMSO) level of theory at 298 K.

Table S1. The relative free energies, ΔG in kJ mol-1, and Boltzmann percentage factors, Bf in %, of the three DHEA conformers at 298 K at the B3LYP-D3BJ/def2-TZVPD, B3LYP-D3BJ/6-31++G (2d,p), and B3LYP-D3BJ/cc-pVTZ levels of theory with the PCM of DMSO.

Conf.	def2-TZVPD		6-31++G(2d,p)		cc-pVTZ	
	**ΔG**	**Bf**	**ΔG**	**Bf**	**ΔG**	**Bf**
DHEA-I	0.0	35.3	0.0	36.7	0.0	34.9
DHEA-II	0.2	32.4	0.2	33.4	0.1	33.4
DHEA-III	0.2	32.2	0.5	29.9	0.2	31.7

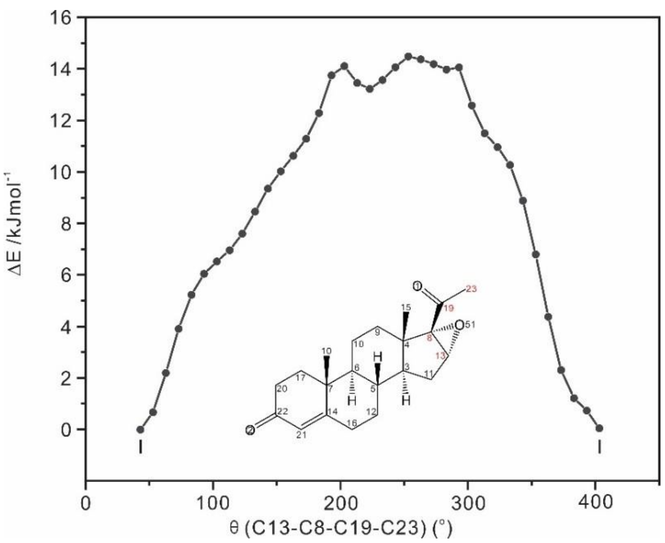

Figure S6. One-dimensional, relaxed potential energy scan of Epoxy-P4 starting from Epoxy-P4-I along the dihedral angle θ (C13-C8-C19-C23) at the B3LYP-D3BJ/def2-TZVP level of theory. The atoms involved in the scan are marked in red in the figure.

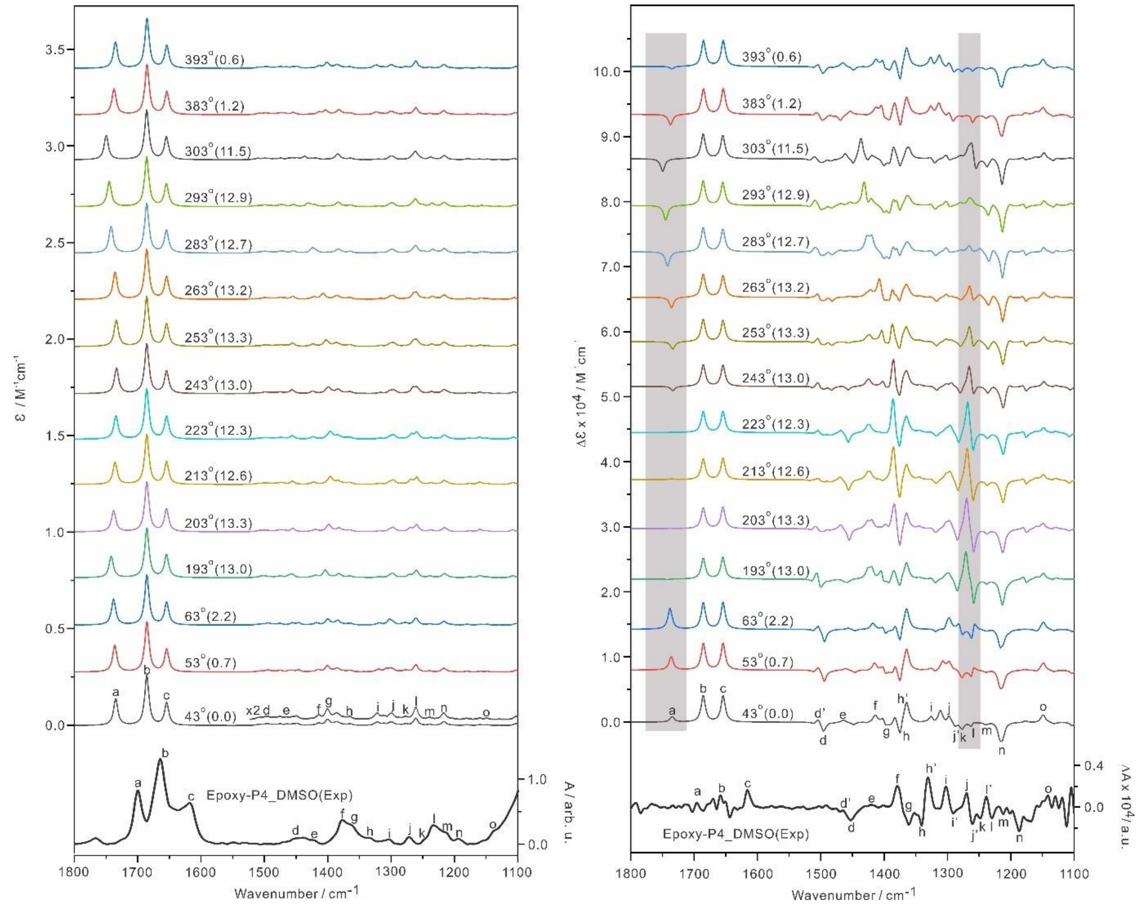

Figure S7. Simulated IR and VCD spectra of Epoxy-P4 at several dihedral angle (C13-C8-C19-C23) values based on the PES scan (Figure S6) at the B3LYP-D3BJ/def2-TZVPD/PCM(DMSO) level at 298 K. The relative free energy in kJ mol^{-1} is listed in the brackets.

Table S2. The relative free energy, ΔG (kJ mol⁻¹) and the Boltzmann population factor, Bf (%) of the three AcO-DHEA conformers at 298 K at the B3LYP-D3BJ/def2-TZVPD level of theory with the PCM of CCl4 and DMSO.

Conformer of AcO-DHEA	DMSO		CCl4	
	ΔG	Bf	ΔG	Bf
AcO-DHEA-I	0.0	52.2	0.5	44.6
AcO-DHEA-II	0.2	47.5	0.0	55.0
AcO-DHEA-III	14.2	0.3	12.7	0.4

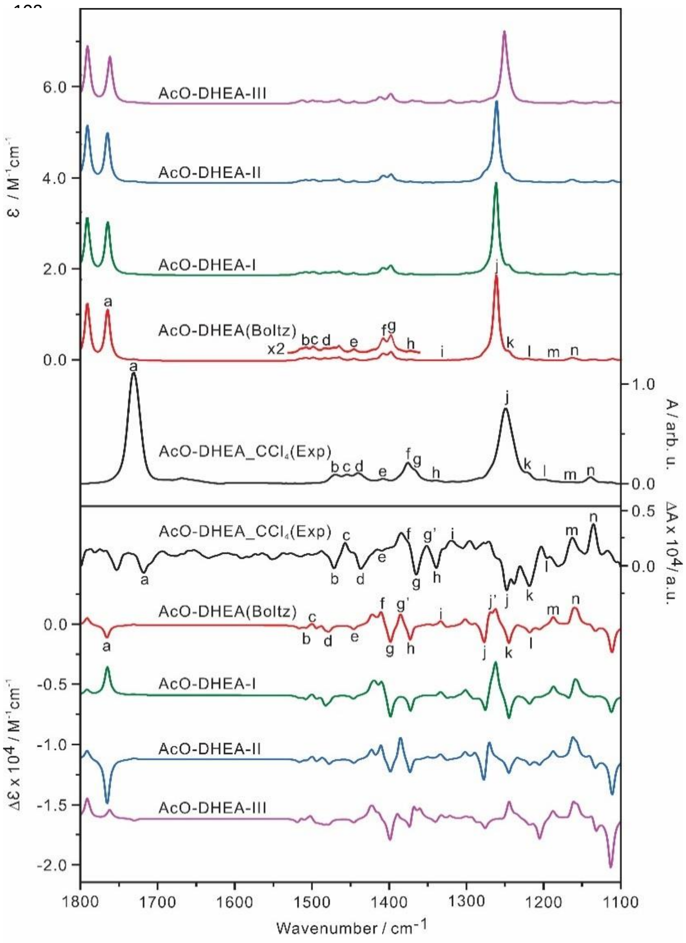

Figure S8. Simulated IR and VCD spectra of the three most stable Aco-DHEA conformers, Aco-DHEA-(I-III) and the related Boltzmann averaged IR and VCD spectra at the B3LYP-D3BJ/def2-TZVPD/PCM(CCl_4) level of theory at 298 K. The experimental units are on the right side. The main IR and VCD band features are labelled as ´a´ to ´n´ to aid 1 comparison.

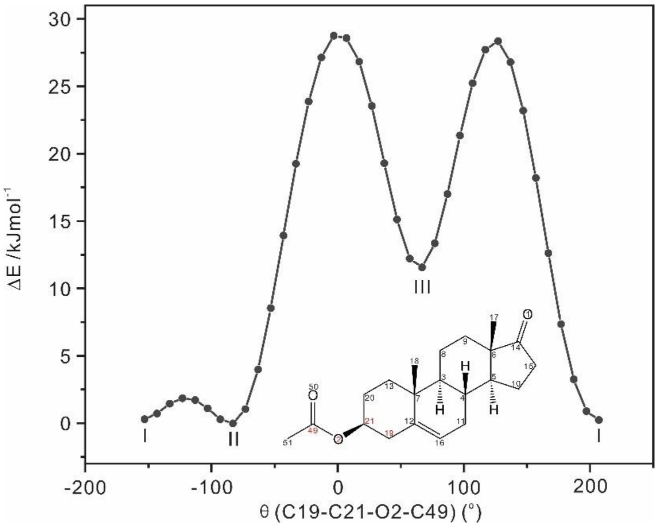

Figure S9. One-dimensional, relaxed potential energy scan of AcO-DHEA starting from AcO-DHEA-I along the dihedral angle θ (C19-C21-O2-C49) at the B3LYP-D3BJ/def2-TZVP level of theory. The atoms involved in the scan are marked in red in the figure.

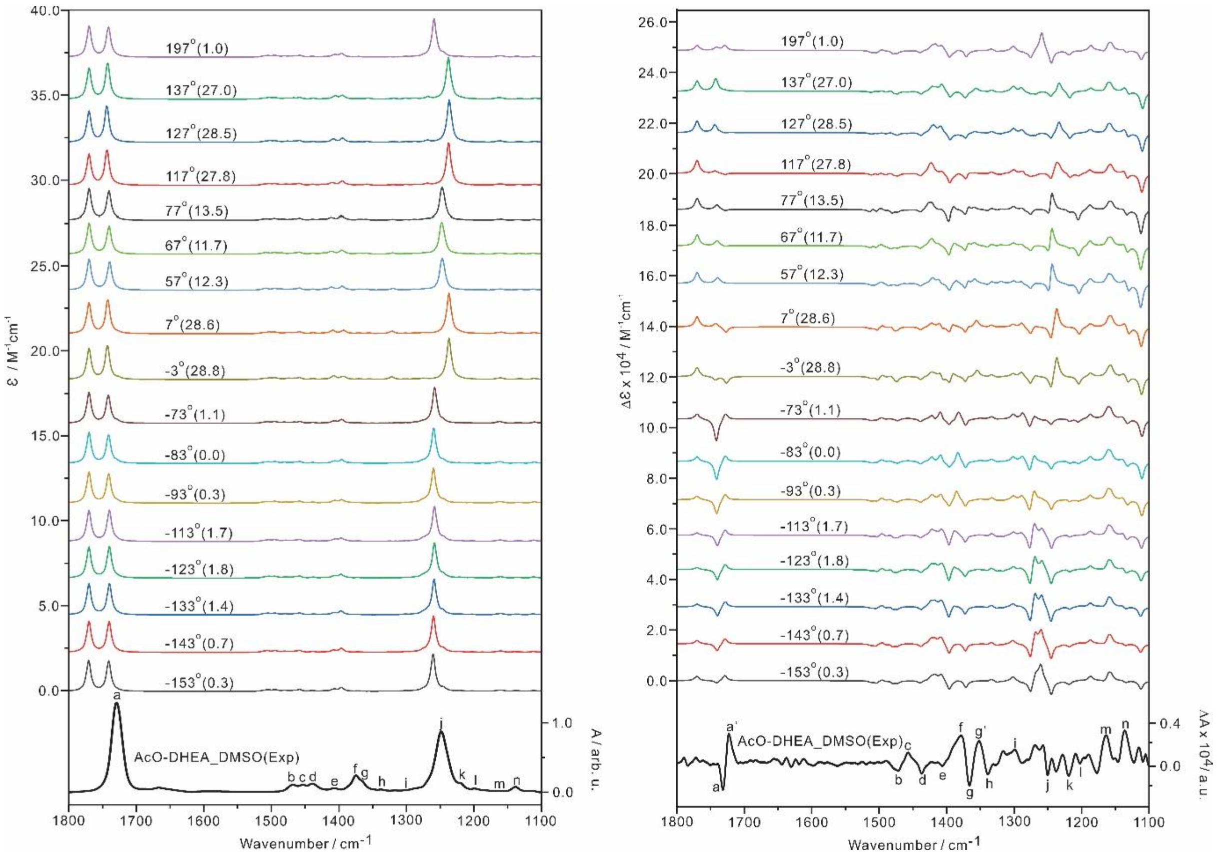

Figure S10. Simulated IR and VCD spectra of AcO-DHEA at several dihedral angle (C19-C21-O2-C49) values along the PES (Figure S9) at the B3LYP-D3BJ/def2-TZVPD/PCM(DMSO) level at 298 K. The relative energies in kJ mol^{-1} are listed in the brackets.

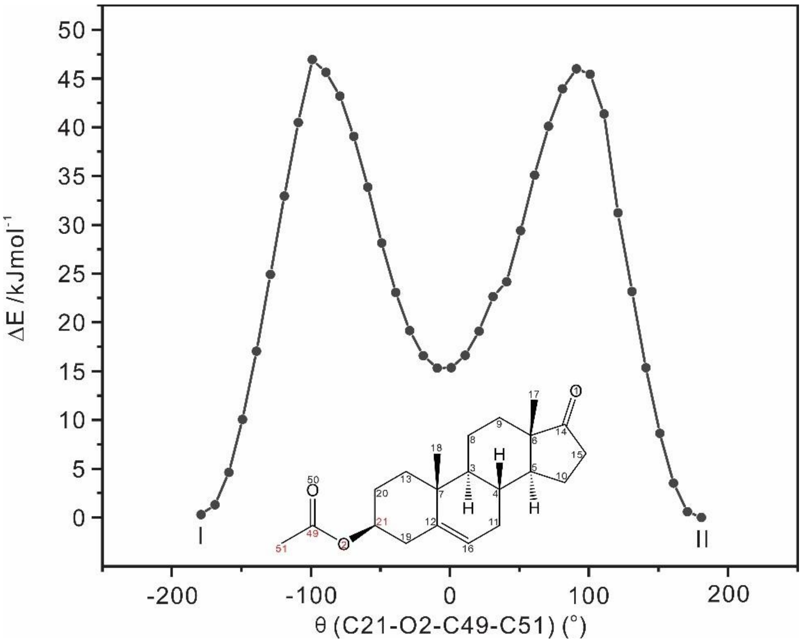

Figure S11. One-dimensional, relaxed potential energy scan of AcO-DHEA starting from AcO-DHEA-I along the dihedral angle θ (C21-O2-C49-C51) at the B3LYP-D3BJ/def2-TZVP level of theory. The atoms involved in the scan are marked in red in the figure.

Point S1:

To evaluate the IR and VCD features in the region above 1700 cm^{-1} of AcO-DHEA in CCl_4, we applied the same approach used for AcO-DHEA in DMSO. We simulated IR and VCD spectra of AcO-DHEA at several selected dihedral angle (C21-O2-C49-C51) (see Figure 8) and (C19-C21-O2-C49) (see Figure S10) values at the B3LYP-D3BJ/def2-TZVPD levels with the inclusion of PCM of CCl_4. The resulting in CCl_4 are shown in Figure S12 and Figure S13.

In Figure S12, the IR and VCD spectra look very similar to the corresponding ones in DMSO which are shown in Figure 8, except that the bands in the >1700 cm^{-1} region experience some blue shift compared to those in DMSO.

In Figure S13, the IR and VCD spectra show similar features as those in DMSO, except in the region >1700 cm^{-1}. In the dihedral angle (C19-C21-O2-C49) values between −153° (around AcO-DHEA-I) and −93° (around AcO-DHEA-II), one can see that the two carbonyl VCD bands located at 1773 cm^{-1} and 1750 cm^{-1} region varies drastically with a slight change in their dihedral angle, going from "+/+" to "−/−"and finally to "+/−". Interestingly, for the values between −123° and −113°, i.e., near the first transition state, the corresponding VCD features were predicted to be "−/−", in good agreement with the observations. We note that the barrier height in the −153° to −93° is under 2 kJ/mol, suggesting that the all these geometries may contribute to the final observed VCD features. To properly predict the VCD features, it is not enough to solely rely on the DFT simulations at the minima. One needs to consider effects of such large amplitude motions, as discussed in Ref. 40 and 66.

Overall, none of the calculations can capture the single IR band observed in the > 1700 cm^{-1} region. This suggests that large amplitude motions are not the cause for the discrepancy between theory and experiment and it is still difficult to capture such IR patterns properly with the DFT calculations presented.

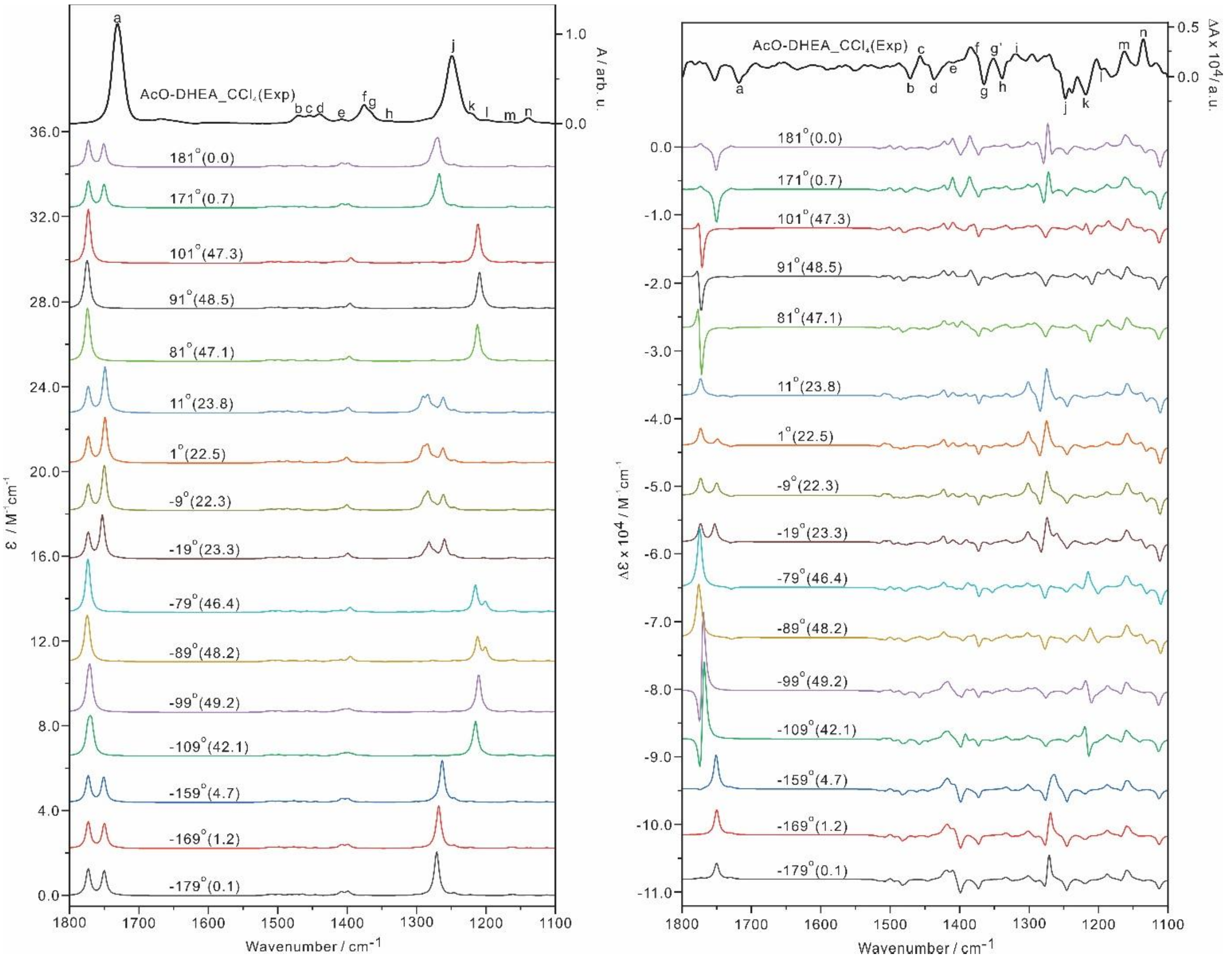

Figure S12. LYP-D3BJ/def2-TZVPD/PCM(CCl_4) level at several dihedral angle values which are based on the potential energy surface scan along the dihedral angle (C21-O2-C49-C51). The relative energies in kJ mol^{-1} are listed in the brackets.

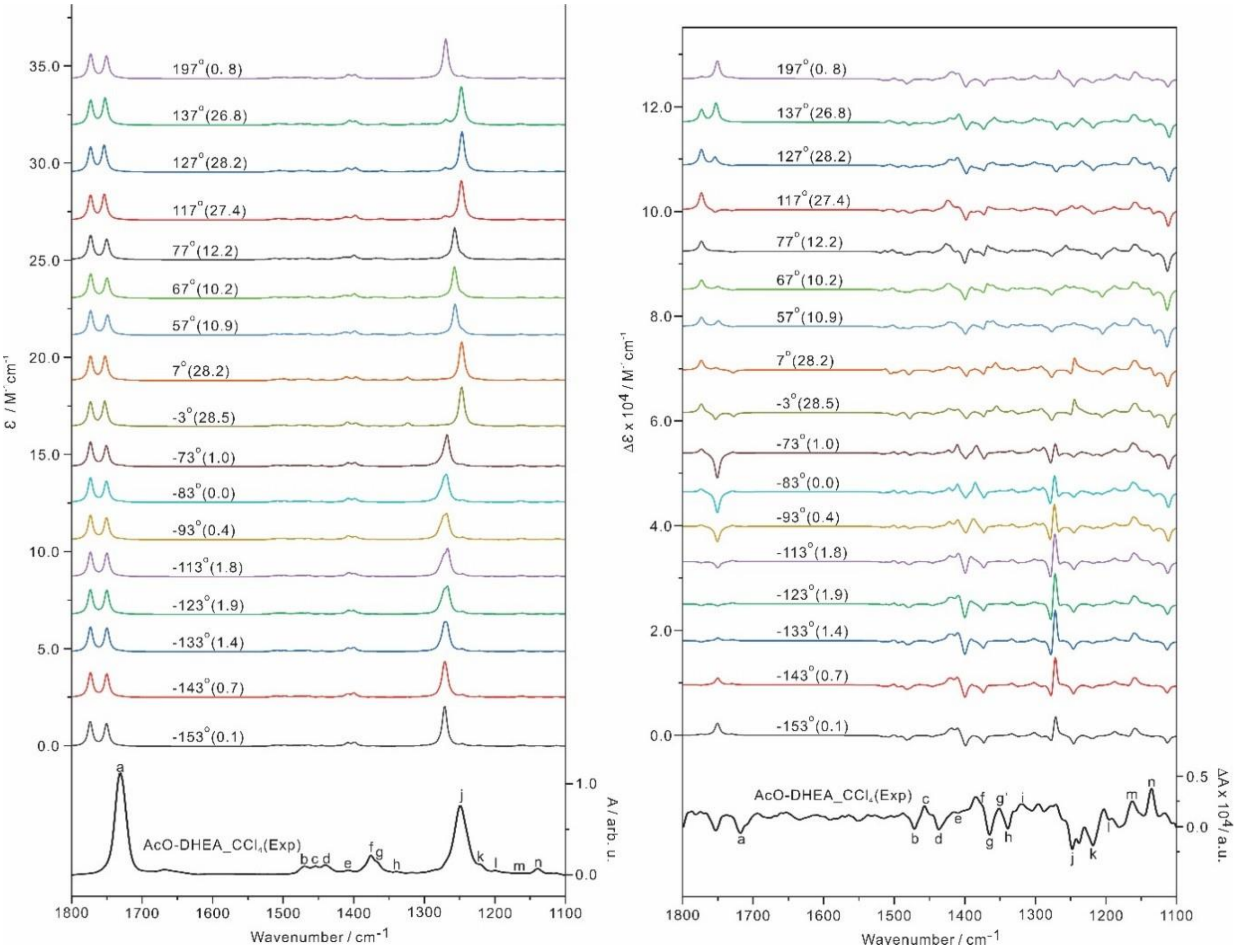

Figure S13. LYP-D3BJ/def2-TZVPD/PCM(CCl_4) level at several 180 dihedral angle values along the potential energy surface scan along the dihedral angle (C19-C21-O2-C49). The relative energies in kJ mol^{-1} are listed in the brackets.

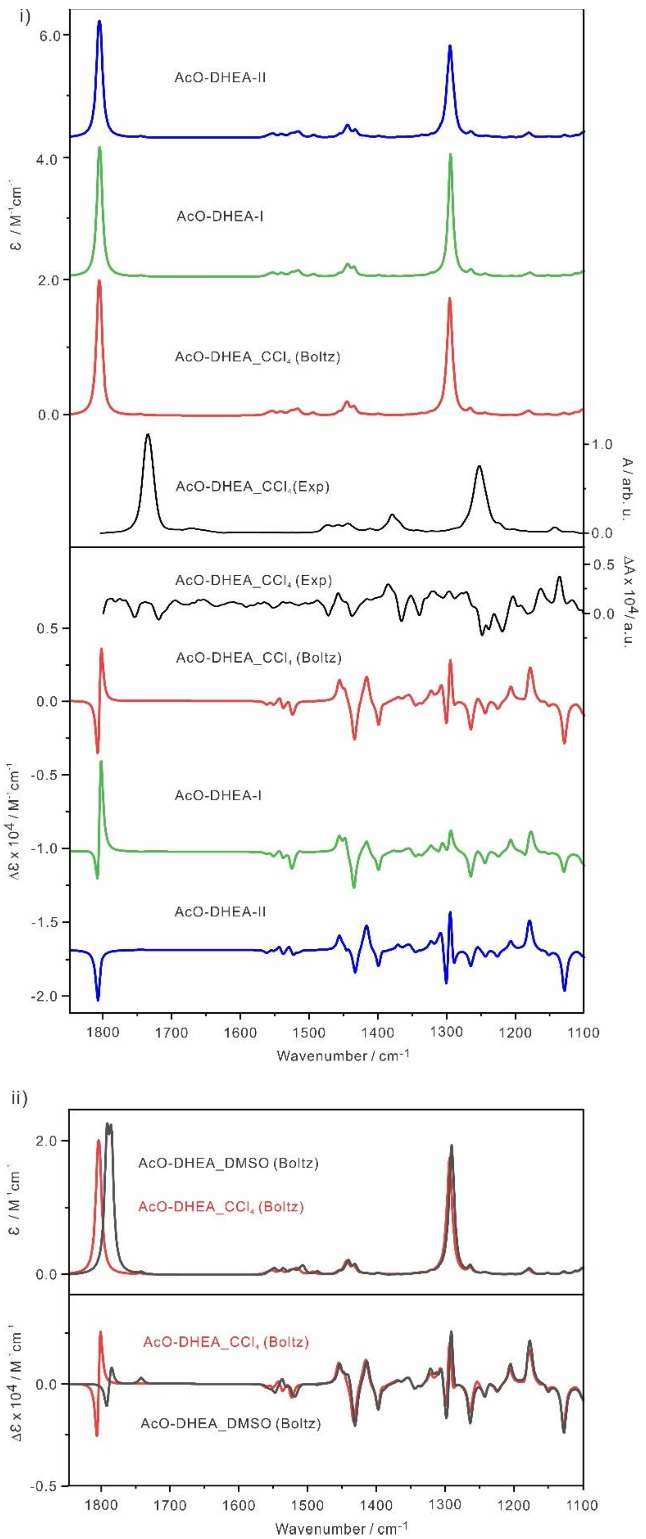

Figure S14. i) LYP-D3BJ/def2-TZVPD//B2PLYP-D3/6-31G(d) with the PCM of CCl_4, as well as the Boltzmann averaged spectra and the experimental spectra. ii) Comparison of the averaged IR and VCD spectra in CCl_4 and in DMSO. The predicted abundances of AcO-DHEA-I (49%) and -II (51%) in CCl_4 differ from AcO-DHEA-1 (51%) and -II (49%)in DMSO.

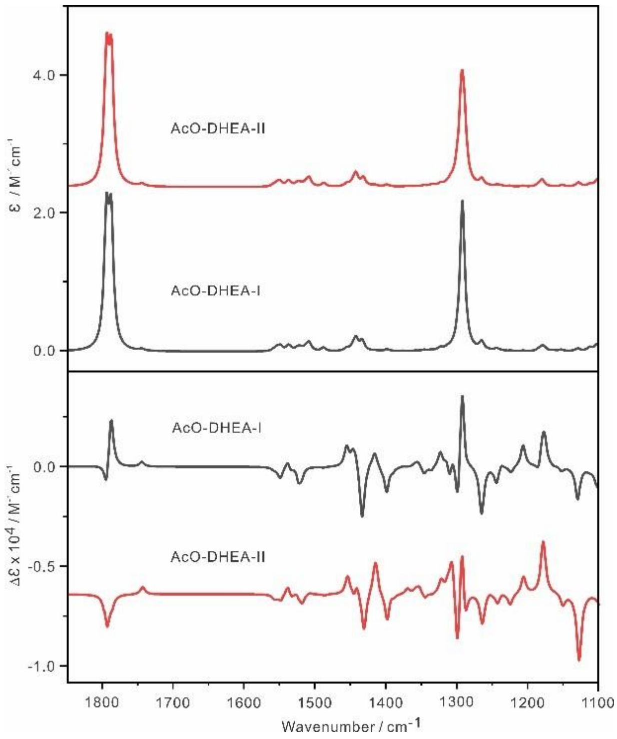

Figure S15. Simulated IR and VCD spectra of AcO-DHEA-I and -II at the B3LYP-D3BJ/def2-TZVPD//B2PLYP-D3/6-31G(d) with the PCM of DMSO.

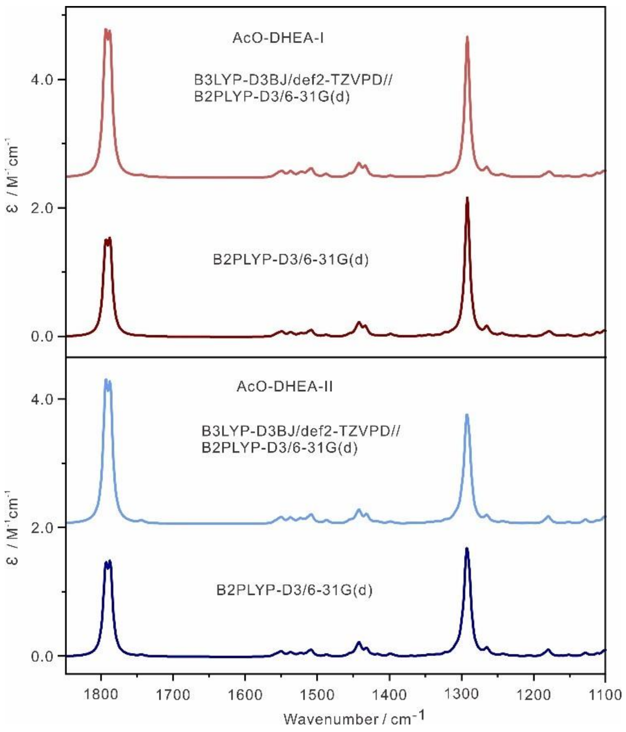

Figure S16. Simulated IR spectra of AcO-DHEA-I (top) and -II (bottom) at the B3LYP-D3BJ/def2-TZVPD//B2PLYP-D3/6-31G(d) versus B2PLYP-D3/6-31G(d) level with the PCM of DMSO.

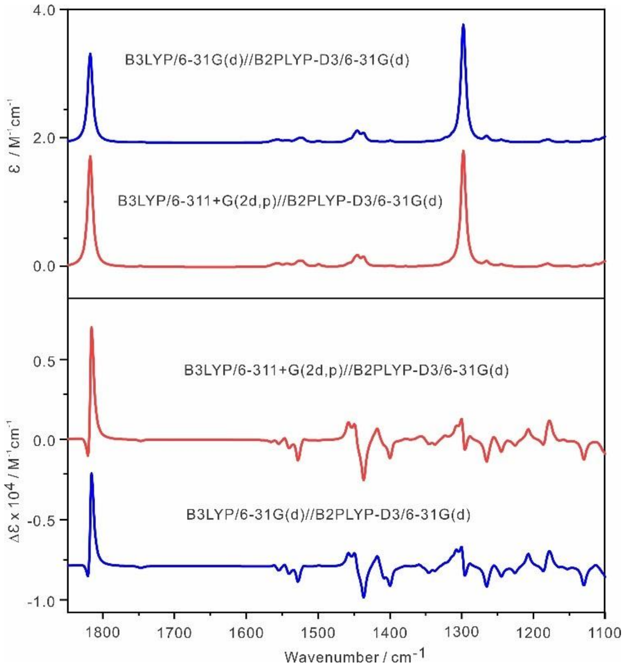

Figure S17. Comparison of the simulated IR and VCD spectra of AcO-DHEA-I at the B3LYP-D3BJ/6-311+G(2d,p)//B2PLYP-D3/6-31G(d)versus at the the B3LYP-D3BJ/6-31G(d)//B2PLYP-D3/6-31G(d) level.
